# Age- and sex-related differences in early-life gut microbiota and intestinal physiology in broiler chickens

**DOI:** 10.1186/s40104-026-01467-y

**Published:** 2026-08-02

**Authors:** Matthias Corion, Muhammad Zeeshan Akram, Ester Arévalo Sureda, Luke Comer, Jeroen Maertens, Natasja Smeets, Nadia Everaert

**Affiliations:** 1 Nutrition and Animal Microbiota EcoSystems (NAMES) Lab, Department of Biosystems, KU Leuven, Kasteelpark Arenberg 30, Heverlee, 3001 Belgium; 2 Kemin Europa NV, Toekomstlaan 42, Herentals, 2200 Belgium

**Keywords:** 16S rRNA sequencing, Barrier function, Host-microbiota crosstalk, Nutrient absorption, Predictive functional profiling

## Abstract

**Background:**

Early-life sex identification technologies are making sex-specific management increasingly feasible in broiler production, yet limited information exists on how males and females differ in the development of their gut ecosystem. While sex-related variation in growth rate and endocrine physiology is well established, much less is known about potential differences in gut morphology, barrier function, microbiota assembly, and intestinal gene expression during the starter period, where early performance divergence between males and females begins to emerge. A clearer understanding of these early-life processes is essential to refine sex-specific nutrition and management strategies. Therefore, this study investigated sex-related differences in gut morphology, intestinal permeability, microbiota composition and predicted functionality, as well as ileal gene expression related to nutrient transport, barrier function, immune response, and metabolic signaling in broilers at 7, 14, and 21 days of age.

**Results:**

Body weight followed a typical early-life pattern and differed between sexes only at d 21, when males were heavier. Gut morphology matured similarly in both sexes, whereas gut permeability declined with age and was lower in males at d 20, suggesting a slightly tighter intestinal barrier. Microbiota structure was predominantly shaped by age, but sex-related divergence emerged with maturation from d 14 onward, especially in the cecum: males were enriched in strict anaerobic fermenters and carbohydrate-degradation/short-chain fatty acid (SCFA)-related pathways, while females showed higher abundance of *Romboutsia*, *Flavonifractor*, and other taxa linked to proteolytic metabolism and the degradation of aromatic amino acid-derived compounds. Gene expression was mainly driven by age, yet consistent sex-specific transcriptional signatures were revealed. Males were more associated with nutrient transport (e.g., *SLC15A1*, *SLC30A1*, *SLC5A1*) and epithelial functional maturation profiles (e.g., *CDX*) over time, whereas females were more associated with tight-junction integrity (e.g., *OCLN*) and amino-acid sensing/transport markers (e.g., *T1R1*, *SLC3A1*). Cecal SCFA concentrations were measured at d 21, yet no differences were found.

**Conclusions:**

Overall, gut development was largely age-driven, but sex-specific differences in barrier function, microbiota composition and function, and epithelial gene expression emerged with maturation, without differences in gut morphology or luminal SCFA concentrations.

**Supplementary Information:**

The online version contains supplementary material available at 10.1186/s40104-026-01467-y.

## Background

In recent years, the poultry sector has increasingly recognized the potential of rearing male and female broilers separately. This trend is partly driven by the increasing availability of commercial post-hatch sexing technologies [1, 2], which allow automated early identification of chick sex shortly after hatching. In parallel, in-ovo sexing is being explored in the broiler industry [3] and is already seeing wider application in the laying-hen sector [4]. As a result, rearing management and feeding strategies can be tailored more precisely to the biological characteristics of each sex.

The rationale for separate-sex rearing lies in the pronounced physiological and metabolic differences between male and female broilers. Males typically exhibit faster growth rates and higher feed intake, whereas females tend to deposit more body fat and mature earlier [5, 6]. Consequently, their nutrient requirements, digestive capacity, and metabolic priorities differ throughout the production cycle. Managing the sexes separately, therefore, enables optimized nutrition and feeding schedules that better match these distinct growth curves [5].

From a production perspective, separate rearing offers several advantages. It improves flock uniformity [7], facilitating more efficient slaughter planning and processing. In practice, male carcasses are typically directed toward cut-up parts, whereas lighter female carcasses are more often marketed as whole birds [8]. Moreover, sex-separate flocks yield a higher proportion of birds meeting their target market-weight categories than mixed-sex flocks [8]. Enhanced uniformity also translates into better feed conversion and lower early and overall mortality [9]. It is also recognized as a welfare indicator, since more uniform flocks reflect more consistent access to feeder and drinker lines [10].

Although the potential of sex-specific rearing for management gains has been demonstrated, a clearer picture of how male and female broilers differ in their gut ecosystems is needed to deepen our understanding and enable more precise nutrition, management, and welfare practices tailored to each sex. Evidence of intrinsic sex differences in broiler gut morphology remains limited. In Ross 308 and Venda chickens, sex effects on villus height (VH), crypt depth (CD), and VH:CD were minimal and largely breed-driven [11]. Similarly, in ducks, gut morphology did not differ between sexes under thermoneutral conditions, whereas chronic heat stress elicited sex-specific intestinal responses, with increased villus width in females and deeper CD and higher goblet-cell density in males [12]. Beyond morphology, sex may also influence gut barrier resilience. Although baseline permeability was comparable between 28-day-old broilers, males exhibited a greater increase in circulating lipopolysaccharide under high stocking density, indicating higher susceptibility to barrier disruption [13].

Beyond morphology and barrier function, emerging evidence also points to sex-dependent gut microbiota profiles and nutrient physiology. Cui et al. [14] reported that in 35-day-old broilers, males exhibited higher cecal abundances of *Bacteroides* and *Lactobacillus*. These taxa were positively associated with host glycan metabolism, while the female-enriched Ruminococcaceae and *Enterococcus* were positively associated with host lipid metabolism [14]. Furthermore, England et al. [5] reported that male broilers generally require higher crude protein than females and that differences in monosaccharide- and amino acid-transporter gene expression may partly underlie performance gaps. However, limited and inconsistent data on sex-specific transporter expression prevent firm conclusions on nutrient absorption differences at this point [5].

Together, these studies suggest that sex-related variation in gut structure, barrier function, microbiota, and nutrient handling may occur in broilers; however, it remains unclear when such differences first emerge during early life under standard production conditions and how host physiological, microbial, and barrier-related parameters co-develop over time. Therefore, the objective of the present study was to characterize male–female differences in (i) gut morphology and intestinal permeability; (ii) microbiota composition and diversity; (iii) microbial functionality, using PICRUSt-based pathway prediction and short-chain fatty acid (SCFA) profiles; and (iv) host physiology, assessed by ileal gene expression, during the starter and early grower phases (d 1–21). This integrated early-life approach allows assessment of whether sex-specific gut differences arise prior to overt performance divergence, providing a foundation for sex-specific nutritional strategies in broiler production.

## Methods

### Animals, housing, and management

**Fig. 1:**
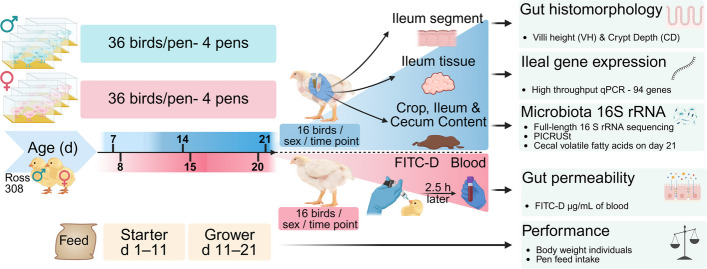
Experimental design and sampling overview. A total of 288 Ross 308 broilers were allocated to eight floor pens (36 birds/pen; four male and four female pens) and fed a starter diet from d 1–11 and a grower diet from d 11–21. Samples were collected on d 7, 14, and 21: ileum for histomorphology and gene expression. At the same time, crop, ileal, and cecal contents were collected for microbiota profiling. Cecal volatile fatty acids were measured on d 21. Gut permeability was assessed by oral fluorescein isothiocyanate-dextran (FITC-D) on a separate set of chickens on d 8, 15, and 20, followed by blood sampling 2.5 h later. Performance was recorded as body weight of sampled birds and pen-level feed intake per dietary phase. (Created with BioRender.com)

A total of 288 one-day-old Ross 308 broilers were obtained from Belgabroed N.V. (Merksplas, Belgium). Birds were assigned in a block design to eight pens (1.3 m^2^; 36 birds per pen; Fig. 1), with four male and four female pens and comparable average start weight. Pens had wood shavings, feeders and drinkers, and were managed with a fully automated climate system. The temperature was set to 34 °C on d 0 and reduced by 0.5 °C per day until it reached 24 °C on d 21. The lighting program was 23 h light and 1 h dark during the first week, and 16 h light, 4 h dark, 2 h light, and 2 h dark from d 7 onward. Feed and water were supplied ad libitum. Mash diets were produced by Research Diet Services BV (Duurstede, Netherlands); the ingredient composition and calculated nutrient contents of the basal starter (d 1–11) and grower (d 11–21) diets are provided in Table S1. On d 2, 7, 8, 14, 15, 20, and 21, body weight was recorded only for the birds selected for sampling (4 per pen and thus 16 per sex), and feed intake was measured at the pen level for the starter and grower phases.

### Chick sampling

On d 7, 14, and 21, 16 birds per sex were sacrificed by electronarcosis followed by decapitation. Immediately after killing, digesta from the crop, ileum, and both ceca were collected into 2-mL tubes, snap-frozen, and stored at −80 °C for microbiota analysis. The ileum was flushed with ice-cold 1 × phosphate-buffered saline (PBS), and tissue from the midpoint was cut into small pieces, snap-frozen in liquid nitrogen, and stored at −80 °C for gene expression analysis. In addition, ileal segments of at least 2 cm from the midpoint were collected after flushing, fixed in 4% formaldehyde for 24 h, and transferred to 70% ethanol for histomorphology.

### Ileal histomorphology

Formalin-fixed ileal tissue was sent to the ImmunoHisto Platform GIGA, Liège University (Liège, Belgium) for paraffin embedding, sectioning, and Alcian Blue–Periodic Acid Schiff staining following standard procedures. Slides were scanned at 20 × and analyzed using NDP.view2 software (Hamamatsu Photonics, Hamamatsu, Japan). For each bird, 15 well-oriented villus–crypt units were measured, including villus height (VH), crypt depth (CD), and the VH:CD ratio.

### In vivo intestinal permeability

On d 8, 15, and 20, a separate set of 16 chickens per sex was randomly selected to assess intestinal permeability, as birds used for this assay were not used for tissue or digesta sampling. This is also indicated by a separate timeline in Fig. 1. At the time of study design, this approach was considered preferable to minimize the potential influence of the oral gavage procedure and associated handling on subsequent sampling. Intestinal permeability was assessed using fluorescein isothiocyanate-dextran (FITC-D; molecular weight 4,000 kDa; Sigma-Aldrich, St. Louis, MO, USA). A FITC-D solution (2.2 mg/mL per bird) was administered orally by gavage, and 1 mL of blood was collected from the jugular vein 2.5 h later. Samples were centrifuged at 3,000 × *g* for 15 min at 4 °C to obtain plasma. Diluted plasma (1:5 in PBS) and standards were pipetted in duplicate into 96-well plates, and fluorescence was measured (Victor3, PerkinElmer, Hopkinton, MA, USA) at 485 nm excitation and 530 nm emission. Absolute FITC-D concentrations were calculated from the standard curve and expressed as µg/mL blood and µg/g body weight (BW).

### Microbiota analysis

#### DNA extraction and full-length 16S rRNA gene amplicon sequencing

Approximately 200 mg of each of the digesta samples was sent to PathoSense (Merelbeke, Belgium) for DNA extraction and purification (ZymoBIOMICS DNA Miniprep Kit, Zymo Research, Irvine, CA, USA). High-quality DNA was used for full-length 16S rRNA gene amplification with primers 27 F (5′-AGAGTTTGATCCTGGCTCAG-3′) and 1492R (5′-GGTTACCTTGTTACGACTT-3′). DNA yield was quantified at each step using a Quantus fluorometer (Promega, Madison, WI, USA), and purity and integrity were checked by NanoDrop spectrophotometry (Thermo Fischer Scientific, Waltham, MA, USA) and agarose gel electrophoresis. Libraries were prepared from the amplicons and sequenced for 24 h on MinION flow cells (R9.4.1) using a GridION platform (Oxford Nanopore Technologies, Oxford, UK).

#### Taxonomic assignment, diversity analysis and multivariate modeling

Raw data were collected and demultiplexed using MinKNOW software (Oxford Nanopore Technologies). Sequencing reads were filtered by quality (Q score > 10), length (1,300–1,700 bp), and yield. Taxonomic assignment was performed with Emu using its default curated database [15]. Microbiome composition and diversity were analyzed in R (version 4.4.3; R Foundation, Vienna, Austria). Bacterial composition was expressed as the relative read abundance of dominant taxa. Alpha diversity (Shannon index) was calculated per sample, visualized as violin plots, and analyzed by two-way ANOVA with main effects of sex, time, and their interaction, followed by Wilcoxon rank-sum tests for pairwise comparisons. Beta diversity was assessed using Bray–Curtis dissimilarities, visualized by principal coordinates analysis (PCoA), and taxa significantly associated with the ordination (*P* < 0.05; 9,999 permutations) were fitted as vectors. Group differences in community structure were tested by permutation ANOVA (PERMANOVA; 9,999 permutations) with assessment of homogeneity of group dispersions, and pairwise PERMANOVA was used for post hoc comparisons. Differentially abundant taxa were identified using Linear Discriminant Analysis Effect Size (LEfSe), retaining taxa with LDA scores > 2.0. Functional profiling of the microbiota was performed using phylogenetic investigation of communities by reconstruction of unobserved states (PICRUSt2), which generated predicted metagenomic pathways from 16S rRNA gene profiles. Predicted pathway abundances were analyzed using Wilcoxon rank-sum tests with Benjamini–Hochberg false discovery rate (FDR) correction (FDR < 0.05) to identify functional pathways differing between groups.

### Ileal gene expression

#### RNA extraction

Total RNA was extracted from frozen ileum tissue using the ReliaPrep™ RNA Tissue Miniprep System (Promega) according to the manufacturer’s instructions. RNA concentration and quality were assessed with a NanoDrop 2000 spectrophotometer (Thermo Fisher Scientific) and 1% agarose gel electrophoresis. cDNA was synthesized from 60 ng total RNA using the Reverse Transcription Master Mix (Standard BioTools, South San Francisco, CA, USA) and analyzed by high-throughput qPCR as described by Akram et al. [16]. Pre-amplification was performed with the PreAmp Master Mix (Standard BioTools), followed by exonuclease I treatment (New England Biolabs, Ipswich, MA, USA).

#### Primer design and validation

A literature-driven ileal gene panel was assembled to capture distinct physiological functions, comprising 12 housekeeping, 23 nutrient transport, 19 barrier function, 18 immune response, 7 endocrine peptide, 5 metabolism-related, 5 oxidative stress-related, and 4 SCFA-related genes. Gene identities, primary functions, and primer sequences are listed in Table S2. Most primers were adapted from Akram et al. [16], with additional validated primers from other studies to broaden coverage of genes relevant to intestinal health and function. All primers were checked in NCBI Primer-BLAST to span exon–exon junctions and avoid off-target amplification. Specificity was confirmed by single-peak melting curves and single, sharply defined agarose-gel bands of the expected size. Assay efficiencies were 90%–110% with *R*^2^ > 0.99, based on three-fold serial dilutions of pooled cDNA on a QuantStudio 6 (Thermo Fisher Scientific). No-template and no-reverse transcription controls were negative, and biological replicates showed low variability (Ct CV < 5%).

#### High-throughput qPCR

Gene expression was analyzed on 96.96 Dynamic Array integrated fluidic circuits (IFCs) using the BioMark™ HD Real-Time PCR System (Standard BioTools) following Akram et al. [16]. For the sample mix, 2.25 µL pre-amplified, Exo I-treated cDNA was combined with 2.5 µL of 2 × SsoFast™ EvaGreen^®^ Supermix (Bio-Rad, Hercules, CA, USA) and 0.25 µL of 20 × DNA Binding Dye (Standard BioTools). The assay mix contained 0.5 µL of each forward and reverse primer (100 µmol/L), 2.5 µL of 2 × Assay Loading Reagent, and 2.25 µL low-EDTA DNA suspension buffer. Sample and assay mixes were loaded onto the IFC according to the manufacturer’s instructions. qPCR was run on the BioMark HD using the GE Fast 96 × 96 PCR + Melt v2 protocol, including a thermal mix step, a hot start at 95 °C for 60 s, 30 cycles of 96 °C for 5 s and 60 °C for 20 s, followed by a melting curve. Non-template controls were included. Quantification cycles (Cq) were obtained with Fluidigm Real-Time PCR Analysis software v4.8.1 (Standard BioTools). Gene expression was normalized to the geometric mean of the three most stable housekeeping genes identified by NormFinder [17]: Glucuronidase beta (*GUS*), Beta-2-Microglobulin (*B2M*), and Lamin B Receptor (*LBR*) at d 7 and 14, and beta-actin (*ACTB*), TAT box-binding protein (*TBP*), and Ribosomal 28S (*r28S*) at d 21. For each target, a standard curve from a pooled reference sample was used for relative quantification, in a dilution series of 1:3 steps including 4–6 standard points.

#### Differential expression and multivariate modeling

Partial least squares–discriminant analysis (PLS-DA) was used to identify gene expression patterns discriminating males from females. Models were built on the gene expression data with leave-one-out cross-validation, and the number of latent variables was selected based on maximum classification accuracy on the cross-validation set. Outliers were evaluated using Q-residuals, Hotelling’s T2, and visual inspection of aberrant profiles. Models were iteratively refined by retaining variables with variable importance in projection (VIP) ≥ 1 and rebuilding the PLS-DA until classification accuracy no longer improved. PLS-DA was performed in PLS Toolbox v9.5 (Eigenvector Research, Wenatchee, WA, USA) in MATLAB v2024b (MathWorks, Natick, MA, USA).

### Cecal volatile fatty acid analysis

SCFAs (acetate, propionate, butyrate, valerate) and branched-chain fatty acids (BCFA; isobutyrate, isovalerate) were measured as described by Akram et al. [18]. Briefly, approximately 250 mg of cecal content was weighed into 2-mL tubes on ice and mixed with 50 μL 2-methylhexanoic acid internal standard and 80 μL of 6 mol/L HCl. Samples were vortexed, kept on ice for 20 min, and extracted with 25% NaCl and tert-butyl methyl ether. After centrifugation at 10,000 × *g* for 5 min at 4 °C, 600 μL of supernatant was transferred to tubes containing anhydrous sodium sulfate, vortexed, and centrifuged again. A 200-μL aliquot was pipetted into screw-neck vials with conical glass inserts and stored at −20 °C until analysis. Volatile fatty acids were quantified on an HP 6890 GC with an automatic sampler, flame ionization detector, and a DB-FFAP capillary column (30 m × 0.32 mm i.d., 0.25 μm film; Agilent Technologies). Nitrogen was used as carrier gas at 25 mL/min, with the column at 130 °C, the injector and detector at 195 °C. SCFA and BCFA concentrations were expressed in mmol/g wet digesta based on external calibration curves.

### Statistical analysis

Body weight, histomorphology, permeability, gene expression, and SCFA/BCFA data were analyzed in R using linear mixed-effects models (*lme4*, *lmerTest*, *nlme*, *emmeans*). Fixed effects were sex, day, and their interaction, with pen as a random effect. Normality and homoscedasticity of residuals were checked using the Shapiro–Wilk test, QQ plots, and Levene’s test. When variances were homogeneous, models were fitted with lmer assuming a single residual variance, and fixed effects were evaluated by Type III tests with Kenward–Roger degrees of freedom [19]. In case of heteroscedasticity, models were fitted with *nlme::lme* using an age-specific residual variance structure, and F-tests used approximate denominator degrees of freedom. For gene expression analyses, *P*-values for fixed effects across the gene panel were adjusted for multiple testing using the Benjamini–Hochberg FDR procedure (FDR < 0.05). Least-squares means were estimated, and simple effects (male vs. female within each day) were compared post hoc using Holm-adjusted pairwise contrasts [20]. Statistical significance was set at *P* < 0.05. Body weight data are presented as mean ± SD to reflect biological variability, and gene expression data are presented as mean ± SE to indicate the precision of group means. Graphs were generated in GraphPad Prism (GraphPad Software, San Diego, CA, USA).

## Results

### Body weight

**Fig. 2:**
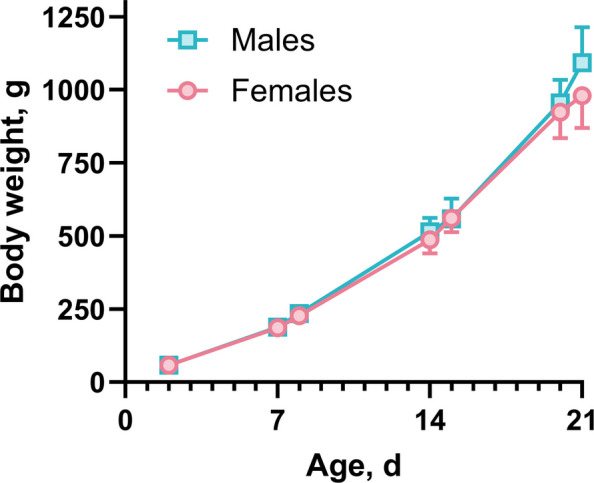
Body weight of male and female broiler chickens that were sampled on different days of age (*n* = 16 per sex per day). Data presented as mean ± SD

Body weight was recorded from the sampled birds on different days during the experiment (Fig. 2). There was a strong effect of day on body weight (*P* < 0.001), whereas the main effect of sex was not significant (*P* = 0.67), and the sex × day interaction only approached significance (*P* = 0.062). Although body weight was comparable between males and females during the early time points, a divergence became apparent by d 21, with males tending to be heavier than females, with 112 g on average. No differences in feed intake between sexes were detected (results not shown).

### Histomorphology analysis

VH, CD, and VH:CD were measured in male and female broilers at different ages (Table S3). As birds aged, VH and CD increased markedly, with VH roughly doubling between d 1 and 21, while VH:CD remained largely unchanged over time. There was a strong effect of age on all three parameters (all *P* < 0.001), whereas no significant effects of sex (VH *P* = 0.622; CD *P* = 0.269; VH:CD ratio *P* = 0.974) or sex × age interaction (all *P* ≥ 0.49) were detected.

### Intestinal permeability

**Fig. 3:**
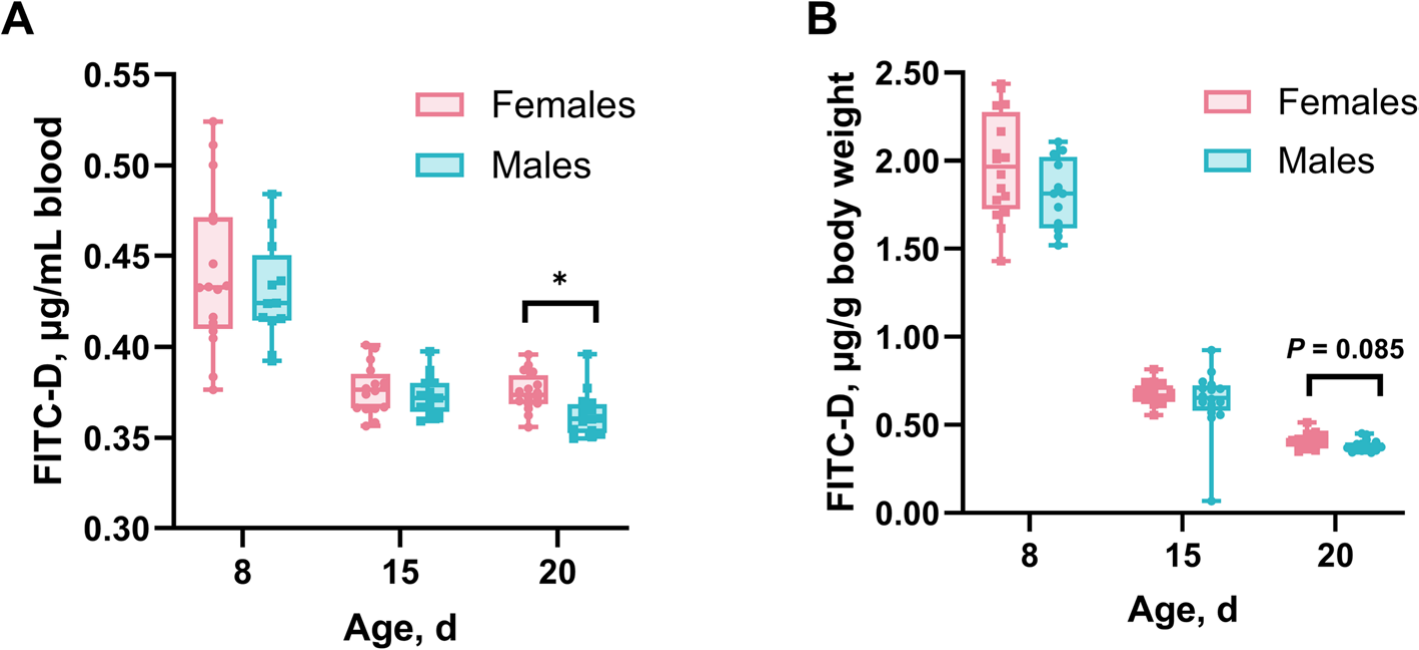
Intestinal permeability of male and female broiler chickens at different ages, measured as fluorescein isothiocyanate-dextran (FITC-D) concentration in (**A**) blood (µg/mL) and (**B**) relative to body weight (µg/g BW). A significant sex effect for the absolute FITC-D concentration in blood was observed on d 20 (*P* = 0.022, Holm-adjusted post hoc test), indicated by * (*P* < 0.05)

FITC-D concentration in plasma (µg/mL) and body weight-corrected FITC-D levels (µg/g BW) were analyzed in male and female broilers at different ages (Fig. 3). There were strong effects of age on both FITC-D concentration (*P* < 0.001) and FITC-D µg/g BW (*P* < 0.001), as values decreased markedly with increasing age. A significant main effect of sex was observed for both variables (FITC-D µg/mL *P* = 0.023; FITC-D µg/g BW *P* = 0.027), whereas the sex × age interaction was not significant (all *P* ≥ 0.29). Across all ages, males consistently showed slightly lower permeability values than females; however, Holm-adjusted post hoc comparisons between sexes within age only identified a significant difference at d 20 for FITC-D concentration in plasma (males lower by 0.013 µg/mL; *P* = 0.022), while the corresponding difference for FITC-D µg/g BW at d 20 did not reach significance (*P* = 0.085), nor did any difference at earlier ages (all *P* ≥ 0.17).

### Microbiota

#### Taxonomic overview

**Fig. 4:**
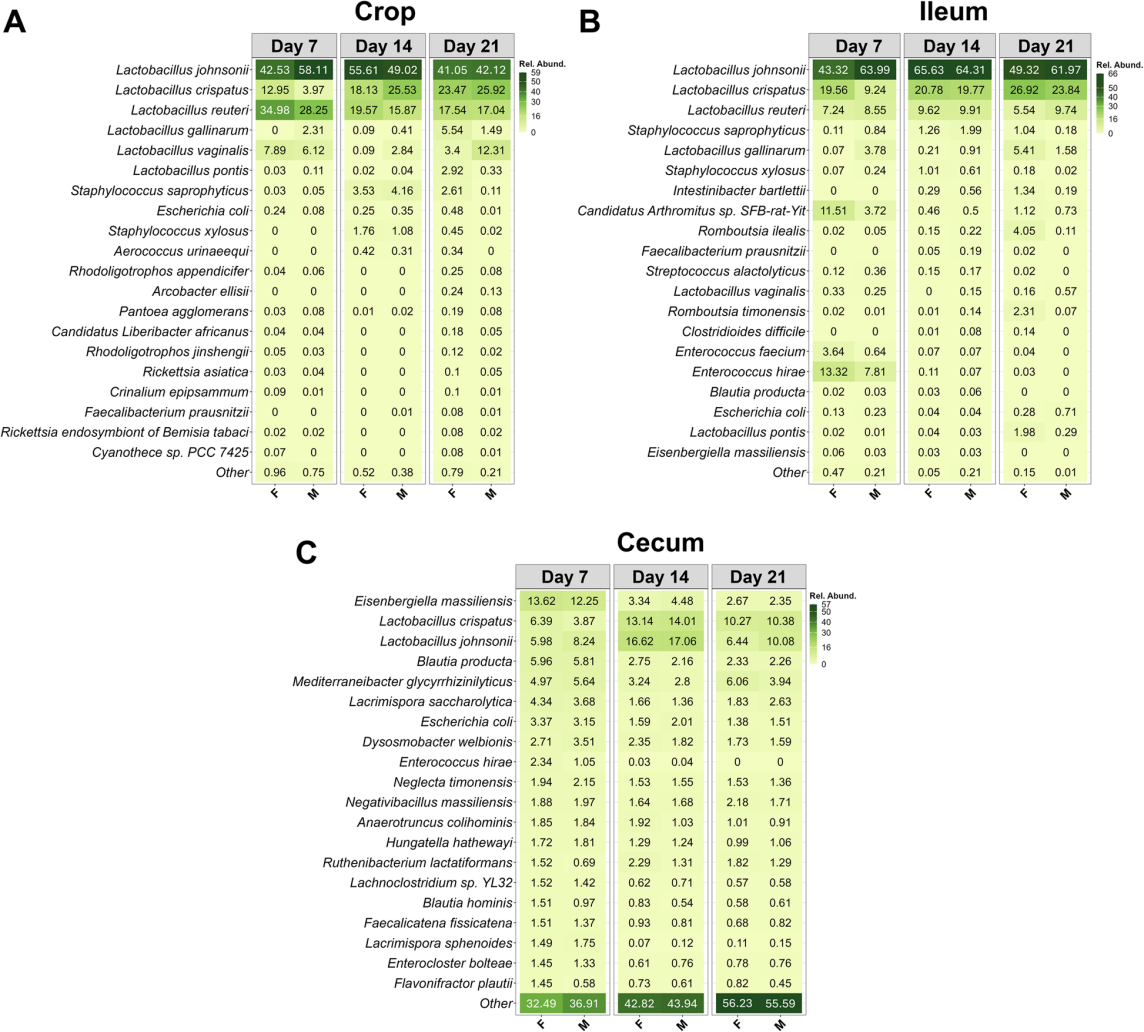
Relative abundance of dominant bacterial species in the crop (**A**), ileum (**B**), and cecum (**C**) of male (M) and female (F) broilers at d 7 (*n* = 16/group), 14 (*n* = 16/group), and 21 (*n* = 16/group). Heatmaps show mean relative abundances (%) of the 20 most prevalent species per segment and time point. All remaining taxa are grouped as “other”

In the crop, at the species level, the microbiota was dominated by *Lactobacillus* species throughout the study (Fig. 4A). On d 7, the most prevalent species were *L. johnsonii* (43%–58%) and *L. reuteri* (28%–35%), followed by *L. crispatus* (4%–13%) and *L. vaginalis* (6%–8%). On d 14, *L. johnsonii* remained the predominant species (49%–56%), with *L. crispatus* (18%–26%) and *L. reuteri* (16%–20%) also abundant. By d 21, *L. johnsonii* continued to dominate (41%–42%), while *L. crispatus* (23%–26%), *L. reuteri* (17%–18%), *L. vaginalis* (3%–12%), and *L. gallinarum* (1%–6%) were consistently detected. Overall, the crop microbiota was largely stable across time points and characterized by the persistent dominance of *L. johnsonii*, *L. reuteri*, and *L. crispatus*.

In the ileum, the microbiota was consistently dominated by *L. johnsonii*, which accounted for 43%–64% on d 7, 64%–66% on d 14, and 49%–62% on d 21 (Fig. 4B). Other abundant taxa included *L. crispatus* (9%–20% on d 7, 19%–21% on d 14, and 23%–27% on d 21) and *L. reuteri* (7%–9% on d 7, 9%–10% on d 14, and 6%–10% on d 21). Minor but consistently detected species were *L. gallinarum* (up to 5% at d 21) and *L. vaginalis* (< 1%). Transient colonizers such as *Enterococcus hirae* (8%–13% at d 7) and *Candidatus Arthromitus* (up to 11% at d 7) were observed early but declined thereafter. By d 21, *Romboutsia ilealis* (up to 4% in females) and *L. pontis* (up to 2%) emerged at low levels.

In the cecum, the bacterial community was more diverse compared with the crop and ileum, with no single species reaching dominance above 20% (Fig. 4C). At d 7, *Eisenbergiella massiliensis* (12%–14%), *Lactobacillus crispatus* (4%–6%), *L. johnsonii* (6%–8%), and *Blautia producta* (6%) were among the most abundant taxa, alongside *Mediterraneibacter glycyrrhizinilyticus* (5%) and *Lacrimispora saccharolytica* (4%). By d 14, *L. johnsonii* increased markedly (16%–17%) and, together with *L. crispatus* (13%–14%), represented the leading cecal species. Other consistently detected genera included *Escherichia coli* (1%–3%), *Dysosmobacter welbionis* (2%–3%), *Neglecta timonensis* (~1.5%), and *Anaerotruncus colihominis* (1.5%–2%). At d 21, *L. johnsonii* (6%–10%) and *L. crispatus* (10%–11%) remained the predominant species, with *E. massiliensis* persisting at 2%–3%. Several taxa, such as *Ruthenibacterium lactatiformans*, *Hungatella hathewayi*, and *Flavonifractor plautii*, remained at low abundances (< 2%) throughout.

#### Alpha and beta diversity

**Fig. 5:**
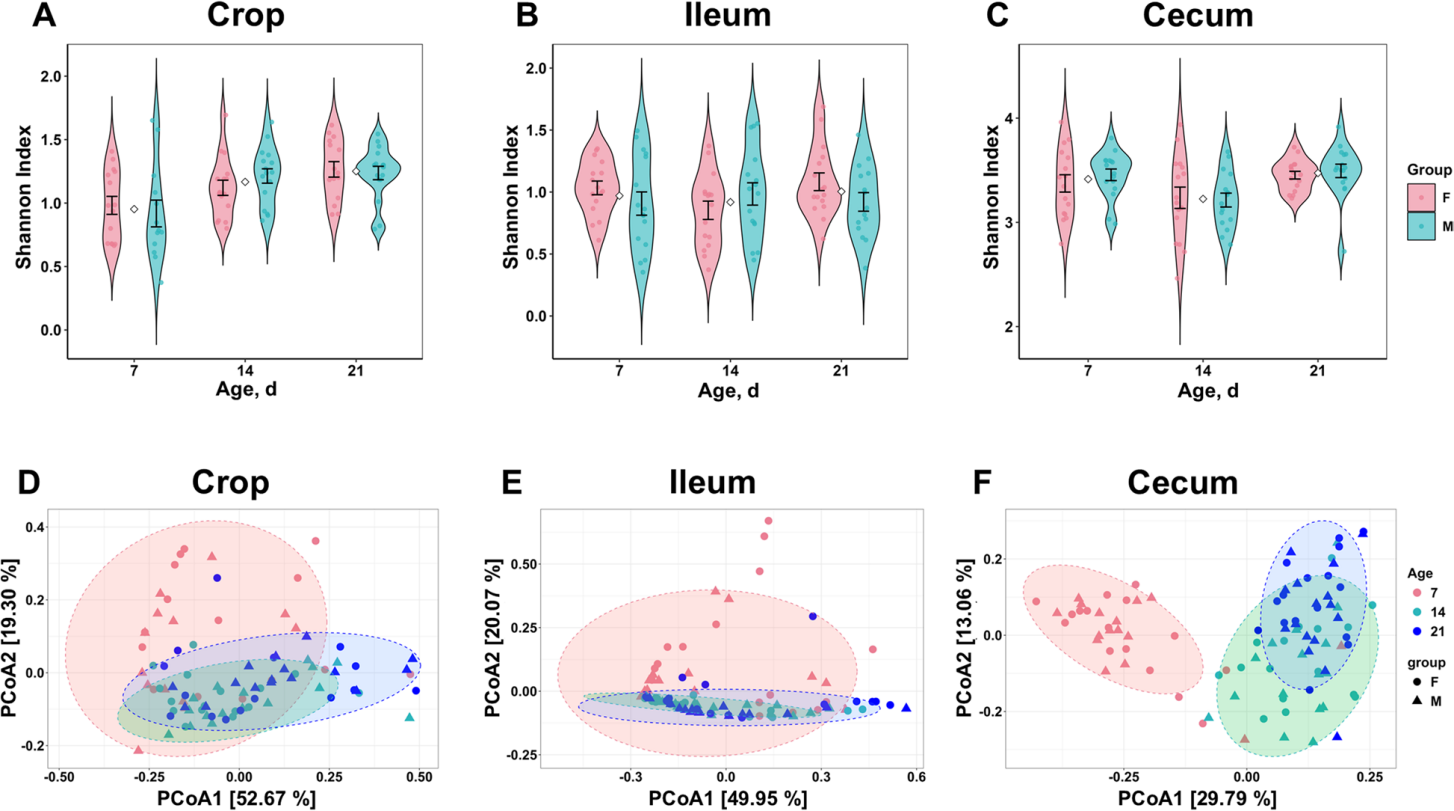
Alpha and beta diversity of the crop (**A** and **D**), ileum (**B** and **E**), and cecum (**C** and **F**) microbiota in male (M) and female (F) broilers at d 7 (*n* = 16/group), 14 (*n* = 16/group), and 21 (*n* = 16/group). **A**–**C** Shannon index values are shown as violin plots with group means ± SE. **D**–**F** Principal coordinates analysis (PCoA) based on Bray–Curtis dissimilarities illustrating age- and sex-related clustering; ellipses represent 95% confidence intervals (*n* = 16/group/timepoint)

Alpha diversity in the crop, assessed by the Shannon index, was significantly influenced by age, showing an increase with age and reaching its peak at d 21 (*P* < 0.001; Fig. 5). In the ileum, neither sex, time, nor their interaction had a significant effect (*P* > 0.05). In contrast, cecal diversity was significantly affected by time (*P* = 0.002), with a decline observed at d 14 followed by a subsequent increase at d 21.

Bray–Curtis analysis demonstrated that microbial communities shifted markedly with age in all gut segments, with the strongest effect observed in the cecum (*P* < 0.001, *R*^2^ = 0.206; Fig. 5). In the crop, both age (*P* = 0.001, *R*^2^ = 0.093) and its interaction with sex (*P* = 0.032, *R*^2^ = 0.011) shaped microbial composition, while sex alone did not. Ileal communities were primarily influenced by age (*P* = 0.005, *R*^2^ = 0.059), with a trend toward sex-related differences (*P* = 0.052, *R*^2^ = 0.026), though no significant interaction was detected.

### Differential abundance of bacteria

**Fig. 6:**
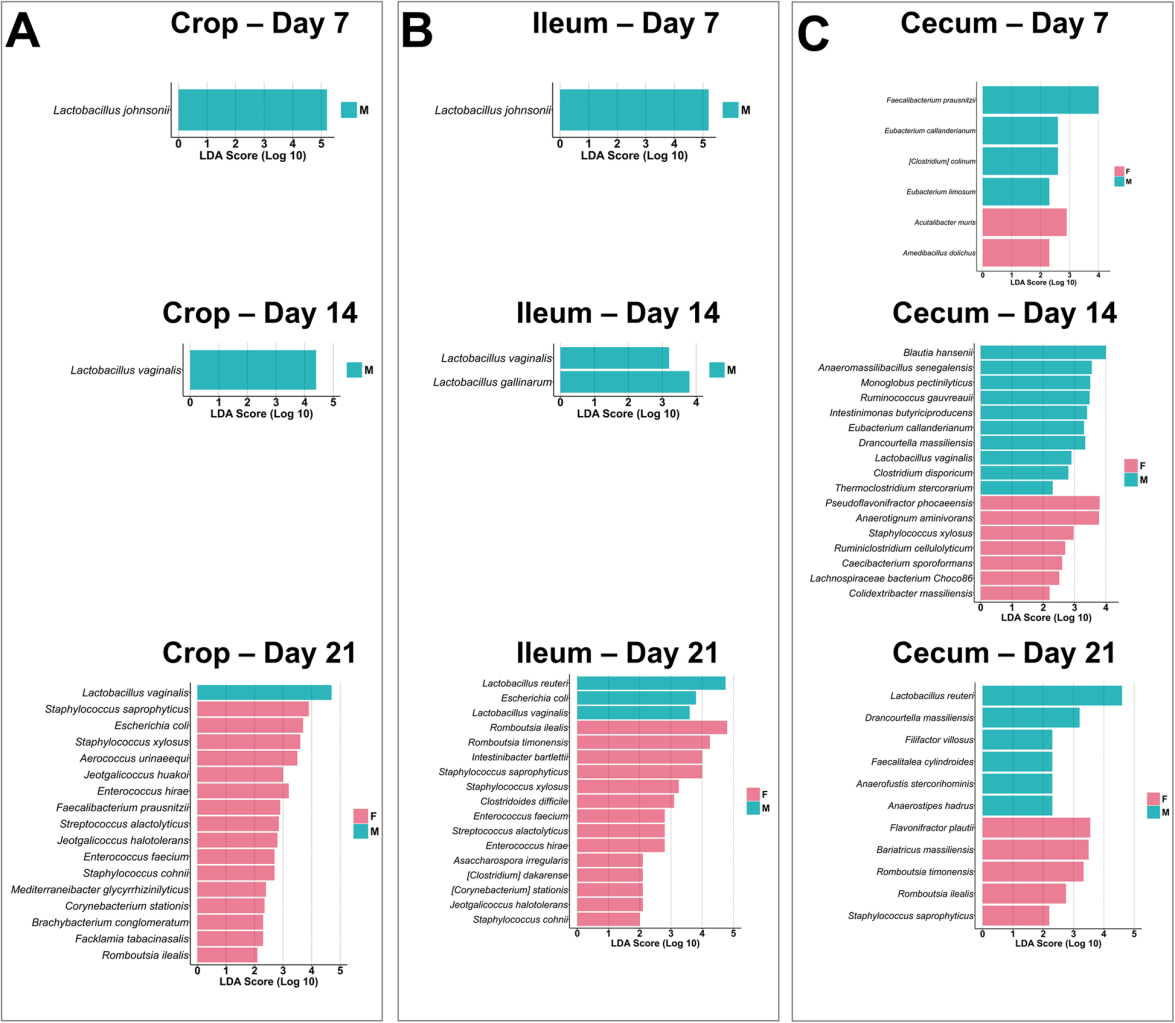
Differentially abundant bacterial species in crop (**A**), ileum (**B**), and cecum (**C**) of male (M) and female (F) broilers at d 7 (*n* = 16/group), 14 (*n* = 16/group), and 21 (*n* = 16/group). Species discriminated by Differential Linear Discriminant Analysis Effect Size (LEfSe) with LDA scores (log10) > 2 are shown. Bar direction indicates enrichment in males or females

In the crop, differential abundance at d 7 and 14 was minimal in both sexes (Fig. 6A). In males, one species was detected at each time point: *L. johnsonii* on d 7 and *L. vaginalis* on d 14 and 21. Females showed no differentially abundant species on d 7 and 14. By d 21, however, female crop communities exhibited broader shifts, with 16 differentially abundant species, including *E. coli*, *Staphylococcus xylosus*, *Staphylococcus alactolyticus*, *Staphylococcus cohnii*, *E. hirae*, *Enterococcus faecium*, and *Faecalibacterium prausnitzii*.

In the ileum, differential abundance at d 7 and 14 was detected only in males (Fig. 6B): *L. johnsonii* was enriched at d 7, whereas *L. vaginalis* and *L. gallinarum* were enriched at d 14. By d 21, ileal communities in males showed enrichment of three species: *L. reuteri*, *L. vaginalis*, and *E. coli*. In contrast, females at d 21 displayed broader ileal shifts, with 14 differentially abundant species, including *S. xylosus*, *S. alactolyticus*, *S. cohnii*, *E. hirae*, *Enterococcus faecium*, *R. ilealis*, and *Romboutsia timonensis*.

In the cecum, sex-specific differences in differential abundance were also evident from early life (Fig. 6C). At d 7, males were enriched in four species: *F. prausnitzii*, *Eubacterium callanderianum*, *[Clostridium] colinum*, and *Eubacterium limosum*, while females showed enrichment of *Acutalibacter muris* and *Amedibacillus dolichus*.

At d 14, males demonstrated higher abundances of ten species, including *Blautia hansenii*, *Monoglobus pectinilyticus*, *Ruminococcus gauvreauii*, *E. callanderianum*, and *L. vaginalis*, whereas females were enriched in seven species such as *Pseudoflavonifractor phocaeensis*, *S. xylosus*, and *Ruminiclostridium cellulyticum*. At d 21, males had six differentially abundant species, including *L. reuteri*, *Drancourtella massiliensis*, *Filifactor villosus*, *Faecalitalea cylindroides*, *Anaerofustis stercorihominis*, and *Anaerostipes hadrus*. Females, on the other hand, were enriched with five species, including *F. plautii*, *Bariatricus massiliensis*, *R. timonensis*, *R. ilealis*, and *Staphylococcus saprophyticus*.

### Microbiota functional profiling

In the crop, few pathways differed between sexes on d 7 (3 pathways) and 14 (4 pathways), whereas a marked divergence was observed at d 21 (46 pathways) (Fig. S1). Early differences included creatine degradation I (males) and menaquinol-8 biosynthesis II (females), while day-21 male-associated pathways were mainly linked to carbohydrate degradation and fermentation, including superpathway of hexitol degradation and pyruvate fermentation to isobutanol, along with amino acid biosynthesis pathways such as L-isoleucine biosynthesis.

In the ileum, functional differences were most pronounced at d 7 (54 pathways), declined at d 14 (13 pathways), and remained evident at d 21 (33 pathways) (Fig. S2). Male-enriched pathways were primarily associated with carbohydrate metabolism and fermentation, including galactose degradation I and heterolactic fermentation, as well as cell wall biosynthesis (peptidoglycan biosynthesis IV). In contrast, female-associated pathways included aromatic amino acid biosynthesis and phospholipid biosynthesis I.

In the cecum, sex differences were limited at d 7 (2 pathways) but increased at d 14 (27 pathways) and d 21 (15 pathways) (Fig. S3). Male-associated functions were dominated by degradation and fermentation processes, including lactose and galactose degradation and acetyl-CoA fermentation to butanoate II, whereas female-associated pathways were more frequently linked to biosynthetic functions, particularly cell wall component biosynthesis.

### Gene expression

Expression of genes related to nutrient transport, barrier function, immune response, endocrine signaling, metabolism, oxidative stress, and SCFA metabolism was analyzed using mixed models including sex, age, and their interaction. Age significantly affected most genes (*P* < 0.01), reflecting expected developmental changes. No main effects of sex or sex × age interactions remained significant after FDR correction (all FDR > 0.05).

**Fig. 7:**
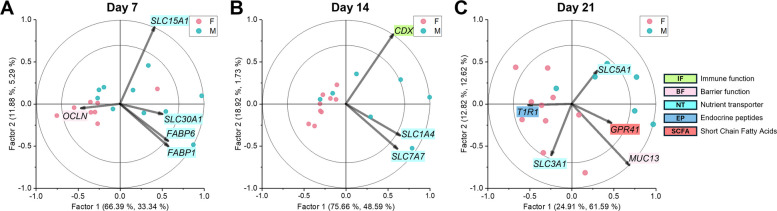
Partial least squares-discriminant analysis (PLS-DA) biplots of ileal gene expression in male (M) and female (F) broilers (n ≈ 10 per sex per day). **A**–**C** PLS-DA models were built separately for d 7, 14, and 21 (cross-validated accuracies: 73.7%, 85.7%, and 86.7%, respectively). Points show individual birds projected onto the first two latent variables; arrows indicate genes with highest contribution (VIP ≥ 1), color-coded by functional category. The percentage of *x* and *y* variance per factor is presented in parentheses, while the outer and inner circles depict the 100 and 50% explained variance, respectively

The univariate findings were further explored using multivariate PLS-DA including all measured genes. The best-performing models contained one latent variable on d 7 (cross-validated accuracy = 73.7%) and d 14 (85.7%), and two latent variables on d 21 (86.7%). For visualization, biplots were generated using the first two latent variables for each day (Fig. 7).

On d 7, sex separation was mainly driven by the nutrient transporters *SLC15A1*, *SLC30A1*, and the fatty acid-binding proteins *FABP1* and *FABP6*, which were associated with males, whereas the tight-junction gene *OCLN* contributed more to female clustering. On d 14, a clearer distinction between sexes emerged, with *CDX*, *SLC1A4*, and *SLC7A7* showing the strongest association with males. By d 21, the classification pattern had shifted, with male samples characterized by higher expression of *SLC5A1, GPR41,* and *MUC13,* while female samples were more associated with *T1R1* and *SLC3A1*. Together, these multivariate results highlight age-dependent molecular signatures associated with sex-specific variation in intestinal gene expression.

### Volatile fatty acid analysis on d 21

Volatile fatty acid concentrations in cecal content were quantified at d 21 to investigate potential sex-related differences in microbial fermentation at the time point when the strongest differences in gut permeability, microbiota composition, and gene expression were observed. Total SCFA and BCFA concentrations did not differ significantly between males and females (Table S4). Similarly, no sex effects were detected for individual major SCFAs, including acetate, propionate, and butyrate (all FDR > 0.66). Individual BCFAs (isobutyrate, isovalerate) also showed no significant sex differences.

## Discussion

### Body weight

Across the early-life period, body weight increased markedly, with only minor sex effects; males were numerically heavier than females only at d 21. This pattern is consistent with previous observations in Ross 308 broilers, where sex differences are minimal during the first two weeks but emerge around d 21, likely reflecting females reaching their growth inflection point earlier than males [7, 21]. In the present study, no significant sex differences in feed intake were observed, although earlier work indicates that male feed intake and performance divergence typically increase from around d 21 onwards [7].

### Broiler gut microbiota

Our study demonstrates pronounced age- and location-dependent structuring and subtle yet biologically meaningful sex-linked variation in the broiler gut microbiota. Across all gut segments, the microbial ecosystem matured from a low-complexity, *Lactobacillus*-dominated community early in life toward a more functionally diversified assemblage by d 21. While age emerged as the principal determinant of microbial composition and diversity, sex-specific differences became increasingly evident with maturation, particularly within the cecum.

### Age-driven stabilization of microbiota

The effect of age was prominent in the crop, where Shannon diversity increased progressively with age. This rapid development of microbial complexity has been widely reported in chickens, with several studies documenting significant rises in community diversity during the first three weeks of life [22, 23]. Beta diversity analysis further emphasized the impact of age, demonstrated by distinct clustering of broiler microbiota at d 7, 14, and 21 across different gut segments, consistent with previous findings [22].

In the crop and ileum of young chicks, the microbiota was dominated by *Lactobacillus* species, particularly *L. johnsonii*, *L. reuteri*, and *L. crispatus*, which maintained remarkably stable proportions from d 7 to d 21. This pattern aligns with chicken gut microbiota studies showing that foregut compartments rapidly become dominated by lactic acid bacteria and reach near-maturity around d 15–28 [22, 24, 25]. The stability of these core taxa suggests early colonization and niche adaptation of *Lactobacillus* in the upper intestine of chicks, forming a persistent “*Lactobacillus* core” that may contribute to acid tolerance, carbohydrate metabolism, nutrient absorption, and competitive exclusion of pathogens [26, 27].

In contrast, the cecal microbiota exhibited higher diversity and higher compositional turnover compared with the crop and ileum, with no single species exceeding 20% relative abundance. This higher microbial diversity is attributed to the unique environment of the cecum, which supports extensive fermentation and prolonged digesta retention, enabling a richer and more complex microbial community [28]. This pattern is consistent with previous observations showing that the cecum becomes increasingly complex over time as cross-feeding fermenters accumulate, and dietary fibre substrates are metabolized [24]. It is important to note that chicks were fed a starter diet from d 1 to 11, followed by a grower diet from d 11 to 21. Although the study was not designed to directly assess dietary effects, these changes in feed composition may have contributed in part to the observed temporal shifts in microbial diversity and community structure.

### Emergence of sex-associated differentiation of the gut microbiota

Although age was the dominant driver of beta diversity, sex-related differences in microbiota composition emerged from d 7 onward. In the upper intestine, males displayed a narrower range of differentially abundant taxa than females, being enriched primarily in *Lactobacillus vaginalis* and *L. reuteri*, consistent with prior findings [29]. Previous studies also indicated that *Lactobacillus* is a gender-differentiated microbe, which may be an important gut microbe relevant to male chickens [29]. The presence of *Lactobacillus* in human or animal males has also been linked to the inhibition of inflammation and increased testosterone levels [30].

Females, by contrast, showed broader enrichment of commensal genera such as *Staphylococcus*, *Enterococcus*, *Faecalibacterium*, and *Romboutsia*. While *Enterococcus* species serve as intestinal commensals and probiotic candidates in poultry, certain strains (e.g., *E. faecalis*, *E. faecium*, *E. hirae*) can act as opportunistic pathogens under specific conditions, impairing growth and gut integrity [31, 32]. Similarly, *Streptococcus* often implicated in secondary infections, has been linked to reduced performance in broilers [33]. Interestingly, *Faecalibacterium*, a saccharolytic, butyrate-producing bacterium, exhibits gut-segment–specific associations with host growth: in the ileum, higher abundance has been associated with impaired intestinal development and increased FCR, indicating reduced growth efficiency [34, 35], whereas in the cecum, *Faecalibacterium* is positively associated with growth performance and body weight [33]. This highlights that the functional impact of a given taxon can differ depending on its intestinal location and local environment. Although *Staphylococcus* species constitute part of the normal gut microbiota, their translocation beyond the gut barrier under stress or poor intestinal development can provoke pathogenicity [36], and their abundance has been negatively correlated with lactate concentration, intestinal morphology, and ileal immune function in chickens [37].

In the cecum, males were enriched early and recurrently with strict anaerobic fermenters such as *Faecalibacterium*, *Eubacterium*, *Ruminococcus*, and *Blautia.* In this compartment, *Faecalibacterium* has previously been reported as a biomarker for enhanced performance due to its positive association with body weight [33]. *Blautia,* previously identified in high-performing broilers [33], generates acetate via acetyl-CoA conversion through the Wood–Ljungdahl pathway, fermenting both glucose and indigestible fibers [38].

Conversely, females exhibited enrichment in *Romboutsia* spp. alongside proteolytic and aromatic-compound metabolizers such as *Flavonifractor* and *Bariatricus* spp. *Flavonifractor plautii*, a well-characterized flavonoid and aromatic-compound degrader, encodes flavone reductases and related enzymes enabling polyphenolic substrate metabolism [39]. *Bariatricus massiliensis*, a recently described Clostridiales member, exhibits fermentative metabolism and genomic potential for amino acid and secondary metabolite turnover [40]. The predominance of these proteolytic and aromatic-compound–degrading taxa suggests a female-associated shift toward amino acid and secondary-metabolite catabolism relative to carbohydrate-centered fermentation, although this was not reflected in differences in BCFA concentrations.

Experimental evidence in mice demonstrates that gonadectomy or hormonal manipulation can shift microbial profiles, supporting a causal link between sex hormones and microbial community structure by influencing gut physiology, immune modulation, and nutrient metabolism [41–43].

### Functional divergence and metabolic specialization

Predicted functional profiles indicate that these sex-specific taxonomic differences translate into divergent metabolic potentials. Male microbiota were consistently enriched in amino acid biosynthesis pathways across gut segments, aligning with the higher metabolic demands of male broiler growth and corroborating previous reports that link microbial nutrient biosynthesis functions to increased weight gain [44]. Carbohydrate degradation and fermentation pathways, including pyruvate-to-butanoate and lysine-to-butanoate fermentation, were consistently overrepresented in the male microbiota across gut segments, indicating a greater capacity to utilize both complex and simple carbohydrates and enhanced SCFA production that supports mucosal health and host energy balance [33]. Consistent with earlier studies, cecal microbiota in males showed enhanced glycan degradation and carbohydrate utilization, increasing energy yield and the availability of carbon skeletons to support host growth, which may contribute to sex-related differences in body weight between male and female broilers. [14]. It should be noted that these predicted functional profiles reflect microbial metabolic potential rather than direct measurements of metabolic output. Notably, despite the enrichment of fermentation pathways in males, cecal SCFA concentrations measured at d 21 did not differ significantly between sexes. This likely reflects the combined influence of microbial production and host absorption and utilization, as luminal SCFA levels are determined not only by microbial synthesis but also by rapid uptake by the intestinal epithelium and systemic metabolism [45, 46]. In line with previous poultry studies, marked shifts in microbiota composition or diversity do not always result in proportional changes in absolute SCFAs [47, 48].

In contrast, female microbiota favored biosynthetic and cell-wall assembly pathways, especially those related to lipid and phospholipid metabolism, consistent with previous studies showing female cecal microbiota involvement in glycerophospholipid metabolism and transportation [14]. The prevalence of these anabolic functions indicates an emphasis on biomass synthesis and cell-surface remodeling. This indicates that female-associated microbiota may prioritize microbial growth and maintenance, reflecting a metabolic orientation distinct from the energy-yielding, fermentation-focused communities observed in males. Importantly, these microbiota-associated functional patterns reflect bacterial metabolic strategies and should not be directly interpreted as indicators of host physiological outcomes.

### Gut morphology and permeability

Consistent with previous reports, we observed no intrinsic sex differences in intestinal morphology under standard production conditions, suggesting that early-life gut structural development is largely age-driven rather than sex-dependent in broilers [11, 12]. Sex-specific alterations in histomorphology have been described under challenge conditions, such as chronic heat stress, where deeper crypts and increased goblet-cell density were reported in male ducks [12].

Gut permeability clearly decreased with age, indicating progressive maturation of the intestinal barrier in early life. Across ages, males tended to exhibit lower plasma FITC-D levels than females, with a significantly lower absolute concentration and a trend toward lower relative FITC-D levels on d 20. Together, these findings suggest that male broilers may develop a slightly tighter intestinal barrier than females by the end of the grower period. As FITC-D permeability is typically interpreted as a relative measure within experiments, it is difficult to directly compare these results to values reported in the literature for broilers.

Literature addressing sex-related differences in gut permeability in broilers is scarce. Nevertheless, within the context of the present study, higher gut permeability is generally linked to increased leakage of antigens and lipopolysaccharides across the mucosa, stronger immune activation, and a diversion of energy and nutrients away from growth [49]. Increased permeability can also enhance epithelial oxygen leakage, favoring facultative bacteria over strict anaerobes and reducing butyrate support to the epithelium [50]. The observed sex-related differences in intestinal permeability were broadly consistent with patterns seen in the microbiota. Males, which exhibited lower plasma FITC-D levels, were enriched in *Lactobacillus* spp. in the upper intestine and strict anaerobic fermenters such as *Faecalibacterium*, *Ruminococcus*, and *Blautia* in the cecum. These taxa are known to associate with carbohydrate fermentation, SCFA production, and maintenance of epithelial integrity [33]. In contrast, females showed greater abundance of facultative and proteolytic taxa, including members of *Staphylococcus* and *Enterococcus,* which are often linked to the production of potentially harmful metabolites, and increased epithelial stress [32, 36].

### Gene expression

Overall, intestinal gene expression was primarily driven by age, reflecting expected developmental maturation of transport, barrier, and metabolic pathways in early life. Sex-related differences were not robust after FDR correction. These observations motivated a multivariate PLS-DA to provide a more integrative perspective, revealing sex-specific molecular signatures that varied with age. At d 7, males were characterized by a stronger association with nutrient transporters (*SLC15A1*, *SLC30A1*) and fatty acid-binding proteins (*FABP1*, *FABP6*), whereas females were more associated with the tight-junction marker *OCLN*. This suggests early differences in molecular signatures related to absorptive versus barrier-oriented processes, with the males showing greater transporter activity for peptides, fatty acids, and zinc, and females emphasizing epithelial integrity. However, this transcriptional orientation did not translate into lower intestinal permeability in females later in life, as indicated by the FITC-D results at d 21. These distinctions are unlikely to affect growth at this stage, but may reflect more efficient nutrient absorption in males, potentially contributing to growth differences later in life [51]. Comparable male-biased nutrient transporter expression has been reported immediately post hatch, although most differences diminished by d 7–14, except for glucose transporters [52].

By d 14, sex separation was more evident, with males characterized by a stronger association with expression of *CDX* and amino-acid transporters (*SLC1A4*, *SLC7A7*). This may reflect differences in amino-acid transport capacity that could support faster protein accretion and increased epithelial differentiation, even though there is no indication that intestinal maturation takes place earlier in males. Future studies combining intestinal gene expression with functional readouts, such as apparent ileal amino acid digestibility, postprandial plasma amino acid profiles, transporter protein abundance, or ex vivo intestinal transport measurements, would help determine whether these molecular patterns translate into sex-specific differences in nutrient absorption. Nutritional studies also point to context-dependent effects, as females – unlike males – upregulated amino-acid transporters under reduced crude-protein diets [53]. These findings suggest that sex differences in nutrient handling are conditional and may become more apparent under specific nutritional demands.

At d 21, the molecular pattern shifted: females showed a stronger association with the expression of genes involved in amino-acid sensing (*T1R1*) and transport (*SLC3A1*), whereas males were characterized by elevated expression of markers for glucose uptake (*SLC5A1*), SCFA signaling (*GPR41*), and mucus barrier support (*MUC13*). Similar sex-specific metabolic routing has been reported in older birds, where males display more glycan-driven metabolism and higher *SLC5A1* expression [14].

Overall, the present findings highlight distinct early-life trajectories in microbiota composition, function, and host physiology between male and female broilers. Future studies extending these observations to later production stages, including slaughter age, would provide valuable insight into the persistence and functional implications of these sex-specific differences under commercial conditions.

## Conclusion

In conclusion, this study demonstrates that sex-related variation in gut microbiota composition, intestinal permeability, and host gene expression emerges early in life and becomes particularly evident by the end of the grower period in broilers. While gut morphology developed similarly in both sexes, males showed a slightly tighter intestinal barrier and a microbial community enriched in fermentative taxa and metabolic pathways associated with carbohydrate degradation and SCFA production. Females, in contrast, exhibited broader enrichment of proteolytic and aromatic-compound–degrading taxa and molecular markers linked to amino acid sensing and epithelial remodeling. Multivariate analyses revealed age-dependent molecular signatures that clearly distinguished males from females. Together, these findings suggest sex-associated differences in early-life gut development, reflected in microbiota composition, predicted function, and host gene expression, with potential implications for nutrient handling, barrier maturation, and host-microbiota interactions. Accounting for sex as a biological factor may therefore support more targeted nutritional and management strategies in broiler production.

## Supplementary Information

Additional file 1: Table S1. Ingredients and calculated nutrient composition of the starter and grower basal diets. Table S2. Primers used for high-throughput qPCR and a brief description of their main functions. There were 81 target genes and 12 reference genes used in total. Table S3. Villus height, crypt depth, and villus height to crypt depth ratioof male and female broiler chickens at different ages, and results of linear mixed model analyses. Table S4. Short-chain and branched-chain fatty acid concentrations in cecum samples of male and female broilers at d 21. Figs. S1–S3. Predicted functional pathways differing between male and female broilers in the crop (Fig. S1), ileum (Fig. S2), and cecum (Fig. S3) at d 7, 14, and 21, based on PICRUSt2.

## Data Availability

The 16S rRNA gene sequencing data can be accessed under Bioproject accession PRJNA1419917 at the NCBI website. Other data will be made available on request.

## Abbreviations

BCFA: Branched-chain fatty acid
BW: Body weight
cDNA: Complementary DNA
CD: Crypt depth
Cq: Quantification cycle
DNA: Deoxyribonucleic acid
F: Female
FCR: Feed conversion ratio
FDR: False discovery rate
FITC-D: Fluorescein isothiocyanate-dextran
GC: Gas chromatography
IFC: Integrated fluidic circuit
LDA: Linear discriminant analysis
LEfSe: Linear discriminant analysis effect size
M: Male
NCBI: National Center for Biotechnology Information
PBS: Phosphate-buffered saline
PCoA: Principal coordinates analysis
PCR: Polymerase chain reaction
PERMANOVA: Permutational multivariate analysis of variance
PICRUSt2: Phylogenetic Investigation of Communities by Reconstruction of Unobserved States 2
PLS-DA: Partial least squares–discriminant analysis
qPCR: Quantitative polymerase chain reaction
rRNA: Ribosomal RNA
SCFA: Short-chain fatty acid
SD: Standard deviation
SE: Standard error
VH: Villus height
VH:CD: Villus height-to-crypt depth ratio
VIP: Variable importance in projection
