# Supplementary material for: Age- and sex-related differences in early-life gut microbiota and intestinal physiology in broiler chickens
Source: J Anim Sci Biotechnol. 2026 Aug 2;17:158. doi: 10.1186/s40104-026-01467-y (PMC13429021; doi:10.1186/s40104-026-01467-y)
Supplement: Supplementary file 1 — Additional file 1: Table S1. Ingredients and calculated nutrient composition of the starter and grower basal diets. Table S2. Primers used for high-throughput qPCR and a brief description of their main functions. There were 81 target genes and 12 reference genes used in total. Table S3. Villus height, crypt depth, and villus height to crypt depth ratioof male and female broiler chickens at different ages, and results of linear mixed model analyses. Table S4. Short-chain and branched-chain fatty acid concentrations in cecum samples of male and female broilers at d 21. Figs. S1–S3. Predicted functional pathways differing between male and female broilers in the crop (Fig. S1), ileum (Fig. S2), and cecum (Fig. S3) at d 7, 14, and 21, based on PICRUSt2. [file 40104_2026_1467_MOESM1_ESM.docx]

**Table S1** Ingredients and calculated nutrient composition of the starter and grower basal diets.

| **Ingredient** (**% as fed)** | **Starter (d 0–11)** | **Grower (d 11–21)** |
| --- | --- | --- |
| ***Ingredient composition (as fed basis, %)*** |  |  |
| Wheat | 50.298 | 50.488 |
| Soybean Meal 48% | 31.180 | 25.830 |
| Rye | 5 | 7.5 |
| Rapeseed | 5 | 7.5 |
| Soybean oil | 4.09 | 4.62 |
| Limestone | 1.51 | 1.41 |
| MCP | 1.36 | 1.27 |
| DL-Methionine | 0.34 | 0.26 |
| L-Lysine HCl | 0.31 | 0.30 |
| Vitamin-Mineral premix | 0.50 | 0.50 |
| Sodium Chloride | 0.21 | 0.22 |
| Sodium bicarbonate | 0.20 | 0.20 |
| L-threonine | 0.19 | 0.13 |
| L-valine | 0.062 | 0.022 |
| ***Calculated nutrient composition (%)*** |  |  |
| Crude protein | 23 | 21.49 |
| Crude fiber | 5.5 | 6.05 |
| Crude fat | 3.05 | 3.21 |
| Starch | 32.32 | 33.73 |
| Calcium | 0.95 | 0.90 |
| Total Phosphorus | 0.73 | 0.71 |
| Available Phosphorus | 0.45 | 0.43 |
| Sodium | 0.15 | 0.15 |
| Chlorine | 0.23 | 0.23 |
| Potassium | 0.97 | 0.90 |
| Lysine dig | 1.25 | 1.15 |
| Methionine + cystine dig | 0.95 | 0.85 |
| Threonine dig | 0.85 | 0.75 |
| Tryptophan dig | 0.24 | 0.23 |
| Isoleucine dig | 0.81 | 0.74 |
| Arginine dig | 1.31 | 1.19 |
| Leucine dig | 1.42 | 1.31 |
| Valine dig | 0.95 | 0.85 |
| AME (kcal) | 2934.99 | 2965.00 |

**Table S2** Primers used for high-throughput qPCR and a brief description of their main functions. There were 81 target genes and 12 reference genes used in total.

| **Function** | **Gene** | **Name** | **Description** | **Primers (‘5 – 3’)** | **Accession number** | **Reference** |
| --- | --- | --- | --- | --- | --- | --- |
| **Barrier function (19)** | **CLDN1** | Claudin-1 | Transmembrane protein of TJ | F: CTTCATCATTGCAGGTCTGTCAG | NM_001013611.2 | Zanu et al., 2020 [1] |
|  |  |  |  | R: AAATCTGGTGTTAACGGGTGTG |  |  |
|  | **CLDN2** | Claudin-2 | Transmembrane protein of TJ | F: ACTGCAGCTGCCCTCGGT | NM_001277622.1 | This study |
|  |  |  |  | R: AAGCTTCACCCTGCTGCTGT |  |  |
|  | **CLDN3** | Claudin-3 | Transmembrane protein of TJ | F: GCCAAGATCACCATCGTCTC | NM_204202.2 | Barekatain et al., 2019 [2] |
|  |  |  |  | R: CACCAGCGGGTTGTAGAAAT |  |  |
|  | **CLDN4** | Claudin-4 | Transmembrane protein of TJ | F: CTGTGCCGGGACACTGAATG | XM_003642382.6 | This study |
|  |  |  |  | R: TCCTCCACAGTGGTGTTTGG |  |  |
|  | **CLDN5** | Claudin-5 | Transmembrane protein of TJ | F: GTCCCAGAAGCGGGAGATAG | NM_204201.2 | This study |
|  |  |  |  | R: CGAGTACTTGACGGGGAAGG |  |  |
|  | **COX-1** | Cyclo-oxygenase 1 | Cyclo-oxygenase 1 | F: GCGCATCAGTAGACCTAGCC | JX160009.1 | Konieczka et al., 2019 [3] |
|  |  |  |  | R: TGGTATTGTGACAGTGCGGG |  |  |
|  | **COX-2** | Cyclo-oxygenase 2 | Cyclo-oxygenase 2 | F:ATTCCTGACCCACAAGGCAC | NM_001167719 | Hollemans et al., 2020 [4] |
|  |  |  |  | R: AGTCAACCCCATGGCCGTAA |  |  |
|  | **CYP450p** | Cytochrome P450 |  | F: ACCACTTCTGGAAGGAGGGA | D49803.1 | Konieczka et al., 2019 [3] |
|  |  |  |  | R: CGCTCTCGTAGACACCCAAC |  |  |
|  | **FABP2** | Fatty Acid Binding Protein 2 | Related with epithelial cell content and occurrence | F: ATGGAAGCAATGGGCGTGAA | NM_001007923.2 | This study |
|  |  |  |  | R: TTCGATGTCGATGGTACGGA |  |  |
|  | **FABP6** | Fatty Acid Binding Protein 6 | Necessary for the transport of bile acids in the gut and it was associated with bacterial presence and inflammation | F: CGGTCTCCCTGCTGACAAGA | XM_046926910.1 | Chen et al., 2016 [5] |
|  |  |  |  | R: CCACCTCGGTGACTATTTTGC |  |  |
|  | **JAM2** | Junctional Adhesion Molecule 2 | Transmembrane protein of TJ | F: GGTACTTGGGGGTCTTCTGC | NM_001397141.1 | This study |
|  |  |  |  | R: TGTGCTTGCAACTAAGAATAGCC |  |  |
|  | **JAM3** | Junctional Adhesion Molecule 3 | Transmembrane protein of TJ | F: CCAGAGTGTTGAGCTGTCCT | XM_417876 | Proszkowiec-Weglarz et al., 2020 [6] |
|  |  |  |  | R: AGAATTTCTGCCCGAGTTGC |  |  |
|  | **LOX-12** | Lipoxygenase 12 | Lipo-oxygenase | F: CTGATTACGCCGTGCTGGAT | XM_015274997.1 | Konieczka et al., 2019 [3] |
|  |  |  |  | R: ATTGGGGCACACAGGAATGT |  |  |
|  | **MUC2** | Mucin 2 | Secretory mucine important in the establishment of the mucus layer | F: CCCTGGAAGTAGAGGTGACTG | XM_046942297.1 | Zanu et al., 2020 [1] |
|  |  |  |  | R: TGACAAGCCATTGAAGGACA |  |  |
|  | **MUC5ac** | Mucin 5ac | Mucin 5ac | F: TGTGGTTGCTATGAGAATGGA | XM_003641322.2 | Forder et al., 2012 [7] |
|  |  |  |  | R: TTGCCATGGTTTGTGCAT |  |  |
|  | **MUC13** | Mucin 13 | Transmembrane mucine that plays a role in cell signalling pathways | F: CCAGGCACCAGAAGTGCTAA | XM_003641585.6 | This study |
|  |  |  |  | R: TGCGTACTGATGCACGTAGT |  |  |
|  | **OCLN** | Occludin | Protein of TJ involved in both inter-membrane and paracellular diffusion of small molecules | F: ACGGCAGCACCTACCTCAA | NM_205128.1 | Zanu et al., 2020 [1] |
|  |  |  |  | R: GGGCGAAGAAGCAGATGAG |  |  |
|  | **ZO-1** | Zonula occludens 1 | Scaffold proteins that form part of the cytoplasmic plaque of TJ | F: ACCACAAGGAGCCATTCCAG | XM_040680624.2 | This study |
|  |  |  |  | R: GTGAGGCCCACACATTACCA |  |  |
|  | **ZO-2** | Zonula occludens 2 | Scaffold proteins that form part of the cytoplasmic plaque of TJ | F: GCCCAGCAGATGGATTACTT | XM_040655419.2 | Barekatain et al., 2019 [2] |
|  |  |  |  | R: TGGCCACTTTTCCACTTTTC |  |  |
| **Endocrine peptides (7)** | **CCK** | Cholecystokinin | Feed intake regulatory hormone | F: GAAGGTAGGGAGCGGCAC | XM_015281332.4 | Song et al., 2012 [8] |
|  |  |  |  | R: TCGGAAAAGGGGGAAAACGA |  |  |
|  | **GHRL** | Ghrelin and Obestatin Prepropeptide | Ghrelin: Hunger hormone, Induces motor activity in the intestinal tract | F: AACTGCTCTGGCTGGCTCT | XM_046926185.1 | He et al., 2018 [9] |
|  |  |  |  | R: CTCCCTCTGTTTCATCTGTAT |  |  |
|  | **Proglucagon** | Proglucagon | Precurose of GLP-1 | F: CACAAGGCACATTCACCAGT |  | Herwig et al., 2020 [10] |
|  |  |  |  | R: TTCTTTGGCAGCTTGACCTT |  |  |
|  | **ProglucagonB** | Proglucagon B | Precursor of GLP-1 | F: CACAAGGCACATTCACCAGT |  | Herwig et al., 2020 [10] |
|  |  |  |  | R: TGGTATTCTCCCAAAAGGTCTC |  |  |
|  | **PYY** | Peptide YY | Peptide tyrosine tyrosine, feed intake regulatory hormone | F: AGGAGATCGCGCAGTACTTCT | NM_001361182.2 | Herwig et al., 2020 [10] |
|  |  |  |  | R: TGCTGCGCTTCCCATACC |  |  |
|  | **T1R1** | Taste 1 Receptor 1 | Taste receptor type 1 member 1 | F: GTGTCATCCCCACAACCAA | XM_425734.4 | He et al., 2018 [9] |
|  |  |  |  | R: CACCACTGCCTCAAAGAAGG |  |  |
|  | **T1R3** | Taste 1 Receptor 3 | Taste receptor type 1 member 3 | F: CATTACCGTCTTCGCCACTC | XM_425740.3 | He et al., 2018 [9] |
|  |  |  |  | R: CTCTGTTCAAATCGGGCTTC |  |  |

**Table S2** (continued)

| **Function** | **Gene** | **Name** | **Description** | **Primers (‘5 – 3’)** | **Accession number** | **Reference** |
| --- | --- | --- | --- | --- | --- | --- |
| **SCFA (4)** | **GPR41** | Growth Associated Protein 41 | FFAR3 – activated by short chain fatty acids | F: GAAGGTGGTTTGGGAGTGAA | XM_427629 | Zhang et al., 2021 [11] |
|  |  |  |  | R: CAGAGGATTTGAGGCTGGAG |  |  |
|  | **GRP43** | Growth Associated Protein 43 | FFAR2 – activated by short chain fatty acids | F: AGGGAATCCGGGATGGAGAA | NM_001318430.1 | This study |
|  |  |  |  | R: ACGCAGTCAGGTTGGTTCAA |  |  |
|  | **GPR84** | Growth Associated Protein 84 | activated by medium-chain and unsaturated long-chain FFAs | F: AACCTCACCTGGCTCAATGG | XM_011538495.3 | This study |
|  |  |  |  | R: GAGCCTATGGAAACTCCGGG |  |  |
|  | **GPR120** | Growth Associated Protein 120 | FFAR4 – activated by medium-chain and unsaturated long-chain FFAs | F: ACTTCACTGCTTTGCCTCAGT | XM_040675455.2 | This study |
|  |  |  |  | R: CCAGTACAAGTGGAGGGTTCA |  |  |
| **Immune response (18)** | **AHSA1** | Activator of HSP90 ATPase Activity 1 | Co-chaperone activator of HSP90 | F: GGGGAAGCCTCCATCAACAA | XM_040672816.2 | This study |
|  |  |  |  | R: TCACTCCTGTGGTCGAGGT |  |  |
|  | **AvBD6** | Avian β-defensin 6 | Avian defense involved in antimicrobial functions and protecting the gut epithelium | F: CTTGCTGTGTGAGGAACAGGTG | NM_001001193.1 | Criado-Mesas et al., 2021 [12] |
|  |  |  |  | R: TTTGGTAGTTGCAGGCAGGAT |  |  |
|  | **AvBD9** | Avian β-defensin 9 | Avian defense with antimicrobial properties and other cellular functions | F: CTGAGACCTCACTGACCACG | NM_001001611.3 | Criado-Mesas et al., 2021 [12] |
|  |  |  |  | R: GTGCTCCCAGGACTCTTCAC |  |  |
|  | **CDX** | Caudal genes | Intestinal tract development | F: ACAGCTGTCCCCTAATGCAC | NM_204676.3 | This study |
|  |  |  |  | R: TCCTTTGTCCTCGTCTTGCC |  |  |
|  | **HSPA4** | Heat Shock Protein Family A member 4 | Member of HSP proteins and play a prominent role in repair and protection of the intestinal environment | F:TGAGACTAATAAATGAATCAACTGCAGT | XM_046927108.1 | Criado-Mesas et al., 2021 [12] |
|  |  |  |  | R: CCCCATATCCACAAAAACAACA |  |  |
|  | **IFNG** | Interferon Gamma | Host defense for combating against the intracellular pathogens including Salmonella | F: ACCTTCCTGATGGCGTGAAG | NM_205149.2 | This study |
|  |  |  |  | R: CTGAAGAGTTCATTCGCGGC |  |  |
|  | **IL1β** | Interleukine 1β | Mediator of the inflammatory response and involved in cellular processes | F: CGCTACACCCGCTCACAGT | XM_046931582.1 | Criado-Mesas et al., 2021 [12] |
|  |  |  |  | R: GCAATGTTGAGCCTCACTTTCTG |  |  |
|  | **IL4** | Interleukin 4 | Cytokine that induces differentiation of naive helper T cells (Th0 cells) to Th2 cells | F: TTATGCAAAGCCTCCACAATTG | XM_046900385.1 | Criado-Mesas et al., 2021 [12] |
|  |  |  |  | R: GTGGGACATGGTGCCTTGAG |  |  |
|  | **IL6** | Interleukin 6 | Humoral immunity related genes | F: CTCGTCCGGAACAACCTCAA | NM_204628.2 | This study |
|  |  |  |  | R: GGAGAGCTTCGTCAGGCATT |  |  |
|  | **IL8** | Interleukin 8 | Secreted in response to pathogenic bacteria infection or specific inflammatory cytokines | F: AGATGTGAAGCTGACGCCAA | NM_205498.2 | This study |
|  |  |  |  | R: GAGCTGAGCCTTGGCCATAA |  |  |
|  | **IL10** | Interleukin 10 | Anti-inflammatory cytokine produced by activated macrophages and T cell | F: CTGAGGGTGAAGTTTGAGGAAAT | NM_001004414.4 | Criado-Mesas et al., 2021 [12] |
|  |  |  |  | R: AGCCAAAGGTCCCCTTAAACTC |  |  |
|  | **IL18** | Interleukin 18 | Pro-inflammatory cytokine, primarily produced by macrophages, targeting T helper type-1 (Th1) cells | F: CTCCTCCACACAGCAACACA | XM_046932259.1 | This study |
|  |  |  |  | R: ATGCAGTTGGCCACTTCTGT |  |  |
|  | **IL22** | Interleukin 22 | Commonly used as marker of inflammation involved in T-lymphocytes activation | F: ACATCAGGGAGAACAACCGC | NM_001199614.1 | This study |
|  |  |  |  | R: TGCCACATCCTCAGCATACG |  |  |
|  | **NOS2** | Nitric oxide synthase-2 | Induce the development of Th1 type of IR in infections | F: CTCCAGCAGAGCTTCTACC TCAA | NM_204961.2 | Criado-Mesas et al., 2021 [12] |
|  |  |  |  | R: GCCAGGTGCTCTTCTATTT TTAATTC |  |  |
|  | **PTGES** | Prostaglandin E synthase | Intestinal inflammatory factor | F: GGCTCTGAGGACAATGCAGA | XM_046928949.1 | This study |
|  |  |  |  | R: CCAGAGGAGAGCACAGCAAA |  |  |
|  | **TLR2** | Toll Like Receptor 2 | Transmembrane receptor for the recognition of gram positive bacteria | F: AGGCGATCCCAAGAGGTTC | XM_046915412.1 | Criado-Mesas et al., 2021 [12] |
|  |  |  |  | R: TTTCCCAAAACATCTGCTGTTG |  |  |
|  | **TLR4** | Toll Like Receptor 4 | Transmembrane receptor for the recognition of gram negative bacteria | F: CAGTCCGTGCCTGGAGGT | NM_001030693.2 | Criado-Mesas et al., 2021 [12] |
|  |  |  |  | R: TTGAGCTTAGCAATTTCAGACTGTTG |  |  |
|  | **TNFa** | Tumor Necrosis Factor a | Regulation of the host immunity against multiple pathogens | F: TTGCGAGGGGAGAGGAGAAA | XM_046927261.1 | This study |
|  |  |  |  | R: GTCAGTACCGCGTCGTCTTT |  |  |

**Table S2** (continued)

| **Function** | **Gene** | **Name** | **Description** | **Primers (‘5 – 3’)** | **Accession number** | **Reference** |
| --- | --- | --- | --- | --- | --- | --- |
| **Nutrient transport (23)** | **ATP1A1** | ATPase Na+/K+ Transporting Subunit Alpha 1 | ATPase Na+/K+ transporting subunit alpha 1 (Calcium transporter) | F- TGCAAATCCATCAGAATCTCGT | XM_046906976.1 | This study |
|  |  |  |  | R- TCCTCATCCAAGGGTTGCAC |  |  |
|  | **CALB1** | Calbindin 1 | Calcium transporter | F- GGCAGGCTTGGACTTAACACC | NM_205513.2 | Zanu et al., 2020 [1] |
|  |  |  |  | R- GTCGGCAACACCTGAGCAAG |  |  |
|  | **FABP** | Fatty Acid Binding Protein | Liver fatty acid binding protein | F: TGAATGTGGCTGGCTCGATTT | AY563636.1 | This study |
|  |  |  |  | R: CAGGTTGACCCCTCCTGTACG |  |  |
|  | **FABP1** | Fatty Acid Binding Protein 1 | Fatty acid binding protein | F: CATCTTCTCTTGTGTTGGGAGC | NM_204192.4 | This study |
|  |  |  |  | R: TGATCATCAGGAAGCCCGAG |  |  |
|  | **SLC1A1** | Solute Carrier Family 1 Member 1 | Excitatory amino acid transporter | F: TGCTGCTTTGGATTCCAGTGT | XM_046936555.1 | Su et al., 2014 [13] |
|  |  |  |  | R:AGCAATGACTGTAGTGCAGAAGTAATATATG |  |  |
|  | **SLC1A4** | Solute Carrier Family 1 Member 4 | Neutral amino acid transporter by ASC system | F: ACAGCAAGCTGTGGTCAGAA | XM_046914471.1 | This study |
|  |  |  |  | R: TCTCCCAGAATGCAATCACAGT |  |  |
|  | **SLC2A1** | Solute Carrier Family 2 Member 1 | Glucose transporter-1 | F: GCAAGATGACAGCTCGCCT | NM_205209.2 | This study |
|  |  |  |  | R: GCTCCTCATATCGGTACAGCC |  |  |
|  | **SLC2A2** | Solute Carrier Family 2 Member 2 | Glucose transporter-2 | F: CAGGAACGTTGGTCCTCTCC | NM_207178.2 | This study |
|  |  |  |  | R: GCGCCCATAGTGTGCTTCTA |  |  |
|  | **SLC2A5** | Solute Carrier Family 2 Member 5 | Transport fructose | F: AAAGAGCTGTAGGTGTGGGC | XM_040689119.2 | This study |
|  |  |  |  | R: CTTTTGCCTGGTTGCCTTCC |  |  |
|  | **SLC3A1** | Solute Carrier Family 3 Member 1 | Protein related to neutral amino acid transporter | F: CCCGCCGTTCAACAAGAG | XM_040667709.2 | Su et al., 2014 [13] |
|  |  |  |  | R: AATTAAATCCATCGACTCCTTTGC |  |  |
|  | **SLC5A1** | Solute Carrier Family 5 Member 1 | Sodium glucose transporter 1 | F: GCCATGGCCAGGGCTTA | XM_046928028.1 | Su et al., 2014 [13] |
|  |  |  |  | R: CAATAACCTGATCTGTGCACCAGTA |  |  |
|  | **SLC5A9** | Solute Carrier Family 5 Member 9 | Transport low concentrations of d-glucose | F: ATACCCAAGGTCATAGTCCCAAAC | XM_040678517.2 | Su et al., 2014 [13] |
|  |  |  |  | R: TGGGTCCCTGAACAAATGAAA |  |  |
|  | **SLC6A19** | Solute Carrier Family 6 Member 19 | Na+-dependent neutral amino acid transporter | F: CCAGAGGGCAATGTAACCCA | XM_040663289.2 | This study |
|  |  |  |  | R: AAGGCTAAGCCGGTTCCTTC |  |  |
|  | **SLC7A1** | Solute Carrier Family 7 Member 1 | Transport lysine, arginine, and histidine | F: CAAGAGGAAAACTCCAGTAATTGCA | XM_046941902.1 | Su et al., 2014 [13] |
|  |  |  |  | R: AAGTCGAAGAGGAAGGCCATAA |  |  |
|  | **SLC7A2** | Solute Carrier Family 7 Member 2 | Transport lysine, arginine, and histidine | F: TGCTCGCGTTCCCAAGA | XM_046916218.1 | Su et al., 2014 [13] |
|  |  |  |  | R: GGCCCACAGTTCACCAACAG |  |  |
|  | **SLC7A5** | Solute Carrier Family 7 Member 5 | Transport hydrophobic amino acids | F: ACGTGCAAGCTCACACCTAA | NM_001030579.3 | This study |
|  |  |  |  | R: CGAGGCCTCCTCAACTCTCA |  |  |
|  | **SLC7A6** | Solute Carrier Family 7 Member 6 | Na+-dependent neutral/cationic amino acid exchanger | F: GCCCTGTCAGTAAATCAGACAAGA | XM_040681080.2 | Su et al., 2014 [13] |
|  |  |  |  | R: TTCAGTTGCATTGTGTTTTGGTT |  |  |
|  | **SLC7A7** | Solute Carrier Family 7 Member 7 | L amino acid transporter 2 | F: CAGAAAACCTCAGAGCTCCCTTT | XM_046911929.1 | Su et al., 2014 [13] |
|  |  |  |  | R: TGAGTACAGAGCCAGCGCAAT |  |  |
|  | **SLC7A9** | Solute Carrier Family 7 Member 9 | Na+-independent neutral/cysteine, cationic amino acid exchanger | F:CAGTAGTGAATTCTCTGAGTGTGAAGCT | XM_046925529.1 | Su et al., 2014 [13] |
|  |  |  |  | R: GCAATGATTGCCACAACTACCA |  |  |
|  | **SLC15A1** | Solute Carrier Family 15 Member 1 | Peptide transporter-1 | F: CAGGATTTCCCTGTGTCAGGT | XM_046906441.1 | This study |
|  |  |  |  | R: GCAGCGTGGACAAGTATGG |  |  |
|  | **SLC30A1** | Solute Carrier Family 30 Member 1 | Efflux of Zn2+ | F: TGGGTGATATGAAGGAC | NM_001389457.2 | This study |
|  |  |  |  | R: AACCTAAGGCATCTCCA |  |  |
|  | **SLC34A2** | Solute Carrier Family 34 Member 2 | n Intestinal phosphate absorption and phosphate homeostasis | F: TGGGGAGAAAGAAGTGTCACAGA | NM_204474.3 | This study |
|  |  |  |  | R: GTGAAGCCACGTTGCCTTTGT |  |  |
|  | **VDR** | Vitamin D Receptor | Transcription factor that mediates the vitamin D3, involved in signalling intestinal calcium and phosphate absorption | F- GCAAAAGGCCGAGAAATGGG | XM_046934191.1 | This study |
|  |  |  |  | R- GAACACCCGTGGCAGATTCA |  |  |

**Table S2** (continued)

| **Function** | **Gene** | **Name** | **Description** | **Primers (‘5 – 3’)** | **Accession number** | **Reference** |
| --- | --- | --- | --- | --- | --- | --- |
| **Metabolism (5)** | **COX16** | Cytochrome Oxidase assembly factor | Enzyme involved in the generation of energy by the mitochondria | F: CCTGCTTTGAAGGAAAAATTGAAG | NM_001197057.2 | Criado-Mesas et al., 2021 [12] |
|  |  |  |  | R: CCAAGTCAGATTGTTCCAATTTCTC |  |  |
|  | **EIF4EBP1** | Eukaryotic Translation Initiation Factor 4E Binding Protein 1 | mTOR pathway proteins—protein synthesis and cell proliferation | F: ATTGAGAACAACCATGTCCAGAAC | XM_040689367.2 | Criado-Mesas et al., 2021 [12] |
|  |  |  |  | R: ATGTCAAACTGCTCTTCTTCACCT |  |  |
|  | **GPX7** | Glutatione peroxidase 7 | Intracellular antioxidant, and plays a great role in the detoxification of various peroxides | F: GGTGCCTCCTTTCCTATGTTCA | NM_001163245.2 | Criado-Mesas et al., 2021 [12] |
|  |  |  |  | R: GTTGGTTCTTCTCCAGTAGAATCAA |  |  |
|  | **mTOR** | mammalian Target of Rapamycin | mTOR pathway proteins—protein synthesis and cell proliferation | F: TGCTGACAAACGCTATGGAGGT | XM_040689168.2 | Criado-Mesas et al., 2021 [12] |
|  |  |  |  | R: AGCCATGACACTGTCCTTATGCT |  |  |
|  | **RPS6KB1** | Ribosomal Protein S6 Kinase B1 | mTOR pathway proteins—protein synthesis and cell proliferation | F: ACACCTGTTGATAGCCCAGATGA | XM_046930143.1 | Criado-Mesas et al., 2021 [12] |
|  |  |  |  | R: GCCACATACGTAAAACCCAGAAA |  |  |
| **Oxidative stress (5)** | **HIF1A** | Hypoxia-Inducible Factor 1alpha | Transcription factor that regulates genes involved in inflammation and cell death | F: CACTTTTTCAGGCAGTTGGAATTG | XM_046917646.1 | Criado-Mesas et al., 2021 [12] |
|  |  |  |  | R: TTTTGCACGCCTTTACACGTT |  |  |
|  | **HMOX2** | Heme Oxygenase 2 | Oxidative stress marker | F: TCCAGTCCACGATGGGAAA | XM_040684168.2 | Criado-Mesas et al., 2021 [12] |
|  |  |  |  | R: GCATTGCCTGCTAGCTTGTCT |  |  |
|  | **LBR** | Lamin B Receptor | Reference gene | F: CTAACCGTCGCTCAGGGC | NM_001396139.1 | This study |
|  |  |  |  | F: TCCAAAAGCAATACCTGGCG |  |  |
|  | **SOD1** | Superoxide Dismutase type 1 | Antioxidant enzyme | F: CCGGCTTGTCTGATGGAGAT | NM_205064.2 | Criado-Mesas et al., 2021 [12] |
|  |  |  |  | R: CTGCGCTGGTACACCCATTT |  |  |
|  | **XDH** | Xanthine Dehydrogenase | Enzyme associated to the synthesis of reactive oxygen species and is member of cellular defence system | F: GAAGCCATTCCATTACTTCAGTTATG | XM_046913189.1 | Criado-Mesas et al., 2021 [12] |
|  |  |  |  | R: AATGTCTGTGCGGATGTTCTTG |  |  |
| **Housekeeping genes (12)** | **18S** | 18S ribosomal RNA | Reference gene | F: ATTCCGATAACGAACGAGACT | XR_006936397.1 | Chen et al., 2016 [5] |
|  |  |  |  | R: GGACATCTAAGGGCATCACA |  |  |
|  | **B-Actin** | Actin Beta | Reference gene | F: TGACTGACCGCGTTACT | NM_205518.2 | This study |
|  |  |  |  | R: GACCCACGATAGATGGGAA |  |  |
|  | **B2M** | Beta-2-Microglobulin | Reference gene | F: TACTCCGACATGTCCTTCAACG | NM_001001750.4 | Borowska et al., 2016 |
|  |  |  |  | R: TCAGAACTCGGGATCCCACTT |  |  |
|  | **GAPDH** | Glyceraldehyde-3-phosphate dehydrogenase | Reference gene | F: CGTGCAGCAGGAACACTA | NM_204305.2 | This study |
|  |  |  |  | R: CAGATCGATGAAGGGATC |  |  |
|  | **GUSB** | Glucuronidase beta | Reference gene | F: GGCAGACTGGTCCTGTTGTTG | NM_001039316.2 | Borowska et al., 2016 |
|  |  |  |  | R: GGGTCCTGAGTGATGTCATTGA |  |  |
|  | **NDUFA** | NADH: Ubiquinone Oxidoreductase | Reference gene | F: TGTGCAGAAACTACAGGACAAACTG | NM_001097637.1 | Criado-Mesas et al., 2021 |
|  |  |  |  | R: AGGGAAAGCTCATTTTCAGCCT |  |  |
|  | **r28s** | Ribosomal 28S | Reference gene | F: GGCGAAGCCAGAGGAAACT | XR_006936395.1 | Borowska et al., 2016 [14] |
|  | **RPS7** | Ribosomal Protein S7 | Reference gene | F: GGCGCTGAGCGAGAAAGG | XM_040667252.2 | This study |
|  |  |  |  | R: CTCCAGGAGAGCCTGGGATA |  |  |
|  | **TBP** | TAT box-binding protein | Reference gene | F: AGCTCTGGGATAGTGCCACAG | XM_046913188.1 | Borowska et al., 2016 [14] |
|  |  |  |  | R: ATAATAACAGCAGCAAAACGCTTG |  |  |
|  | **TUBAT** | Tubulin alpha Chain | Reference gene | F: CAAGCATGAATGCCAACTCTCC | NM_205444.2 | This study |
|  |  |  |  | R: TCACGCATGGTTCGTCCT |  |  |
|  | **UB** | Ubiquitin B | Reference gene | F: GGGATGCAGATCTTCGTGAAA | X02650.1 | De Boever et al., 2008 [15] |
|  |  |  |  | R: CTTGCCAGCAAAGATCAACCTT |  |  |
|  | **YWHAZ** | Tyrosine 3-monooxygenase 5-monooxygenase activation protein | Reference gene | F: GCAAGCAGAAAGCAAAGTTTTCT | XM_046911632.1 | Criado-Mesas et al., 2021 [12] |
|  |  |  |  | R: TGTGATTGCTCCACAATCCCT |  |  |

**Table S3** Villus height, crypt depth, and villus height to crypt depth ratio (VH:CD) of male and female broiler chickens at different ages, and results of linear mixed model analyses (Sex × Age, Pen as random effect).

| Age (days) | **Males** | | | **Females** | | | 2-way ANOVA heteroscedastic mixed model  Sex *P* = 0.622 / **Age *P* < 0.0001 /** Sex*Age *P* = 0.491  Pen (random) LRT *P* = 0.066; ICC = 0.214  Multiple unpaired Holm-adjusted t-test *P*-values: |
| --- | --- | --- | --- | --- | --- | --- | --- |
|  | mean | SD | N | mean | SD | N |  |
| **Villus Height**  **(μm)** |  |  |  |  |  |  |  |
| 1 | 303.4 | 38.9 | 8 | 293.2 | 37.9 | 8 | 0.294 |
| 3 | 355.0 | 40.5 | 7 | 355.3 | 40.0 | 8 | 0.481 |
| 7 | 452.3 | 60.8 | 16 | 451.9 | 40.7 | 15 | 0.997 |
| 14 | 542.8 | 36.6 | 15 | 535.8 | 73.1 | 16 | 0.833 |
| 21 | 666.0 | 103.6 | 14 | 625.5 | 63.1 | 14 | 0.302 |
| **Crypt Depth**  **(μm)** |  |  |  |  |  |  | 2-way ANOVA Kenward-Roger Type III  Sex *P* = 0.269 / **Age *P* < 0.0001 /** Sex*Age *P* = 0.731  Pen (random) LRT *P* = 0.233; ICC = 0.063  Multiple unpaired Holm-adjusted t-test *P*-values: |
| 1 | 56.7 | 10.1 | 8 | 57.3 | 7.1 | 8 | 0.879 |
| 3 | 81.5 | 12.4 | 7 | 80.9 | 6.9 | 8 | 0.865 |
| 7 | 106.1 | 13.6 | 16 | 101.4 | 8.7 | 15 | 0.326 |
| 14 | 119.2 | 12.0 | 15 | 111.7 | 12.8 | 16 | 0.116 |
| 21 | 122.0 | 15.0 | 14 | 115.6 | 10.0 | 14 | 0.184 |
| **VH:CD ratio** |  |  |  |  |  |  | 2-way ANOVA Kenward-Roger Type III  Sex *P* = 0.974 / **Age *P* < 0.0001 /** Sex*Age *P* = 0.510  Pen (random) LRT *P* = 0.064; ICC = 0.115  Multiple unpaired Holm-adjusted t-test *P*-values: |
| 1 | 5.5 | 1.0 | 8 | 5.1 | 0.7 | 8 | 0.223 |
| 3 | 4.4 | 0.5 | 7 | 4.4 | 0.6 | 8 | 0.806 |
| 7 | 4.3 | 0.7 | 16 | 4.5 | 0.4 | 15 | 0.554 |
| 14 | 4.6 | 0.5 | 15 | 4.8 | 0.4 | 16 | 0.398 |
| 21 | 5.5 | 0.6 | 14 | 5.4 | 0.6 | 14 | 0.987 |

Note: Linear mixed models included sex, age, and their interaction as fixed effects and pen (nested within sex) as a random effect. Residual variance homogeneity across ages was assessed using Levene’s test. For villus height, evidence of heteroscedasticity (Levene *P* = 0.0016) led to the use of a heteroscedastic mixed model with age-specific residual variances; crypt depth and VH:CD ratio met the equal-variance assumption and were analyzed with homoscedastic models. *P*-values for fixed effects were obtained using Type III ANOVA (Kenward–Roger where applicable). Random-effect significance was assessed by likelihood ratio test (LRT); the intraclass correlation coefficient (ICC) indicates the variance proportion explained by pen. Pairwise Male–Female comparisons within each age were adjusted by Holm’s method.

**Table S4** Short-chain and branched-chain fatty acid concentrations in cecum samples of male and female broilers at day 21 (µmol/g wet digesta).

| **SCFA concentration (µmol/g)** | **Males** | | | **Females** | | | **Raw *P*-values** | **Holm-adjusted t-test *P*-values** |
| --- | --- | --- | --- | --- | --- | --- | --- | --- |
|  | **Mean** | **SD** | **N** | **Mean** | **SD** | **N** |  |  |
| Acetate | 106.63 | 29.35 | 15 | 110.75 | 31.18 | 16 | 0.76 | 0.93 |
| Propionate | 15.13 | 7.06 | 15 | 14.36 | 5.55 | 16 | 0.77 | 0.93 |
| Butyrate | 22.25 | 7.97 | 15 | 21.75 | 10.39 | 16 | 0.94 | 0.94 |
| Valerate | 2.00 | 0.50 | 15 | 1.75 | 0.36 | 16 | 0.16 | 0.66 |
| **BCFA concentration (µmol/g)** |  |  |  |  |  |  |  |  |
| Isobutyrate | 1.62 | 0.45 | 15 | 1.46 | 0.30 | 15 | 0.31 | 0.66 |
| Isovalerate | 1.44 | 0.40 | 15 | 1.26 | 0.43 | 16 | 0.33 | 0.66 |

Note: SCFA, short-chain fatty acid; BCFA, branched-chain fatty acid. Sex differences were analyzed using linear mixed-effects models with sex as a fixed effect and pen as a random intercept. Reported *P*-values are Holm-adjusted for multiple testing. All data met the assumption of homoscedasticity.

.


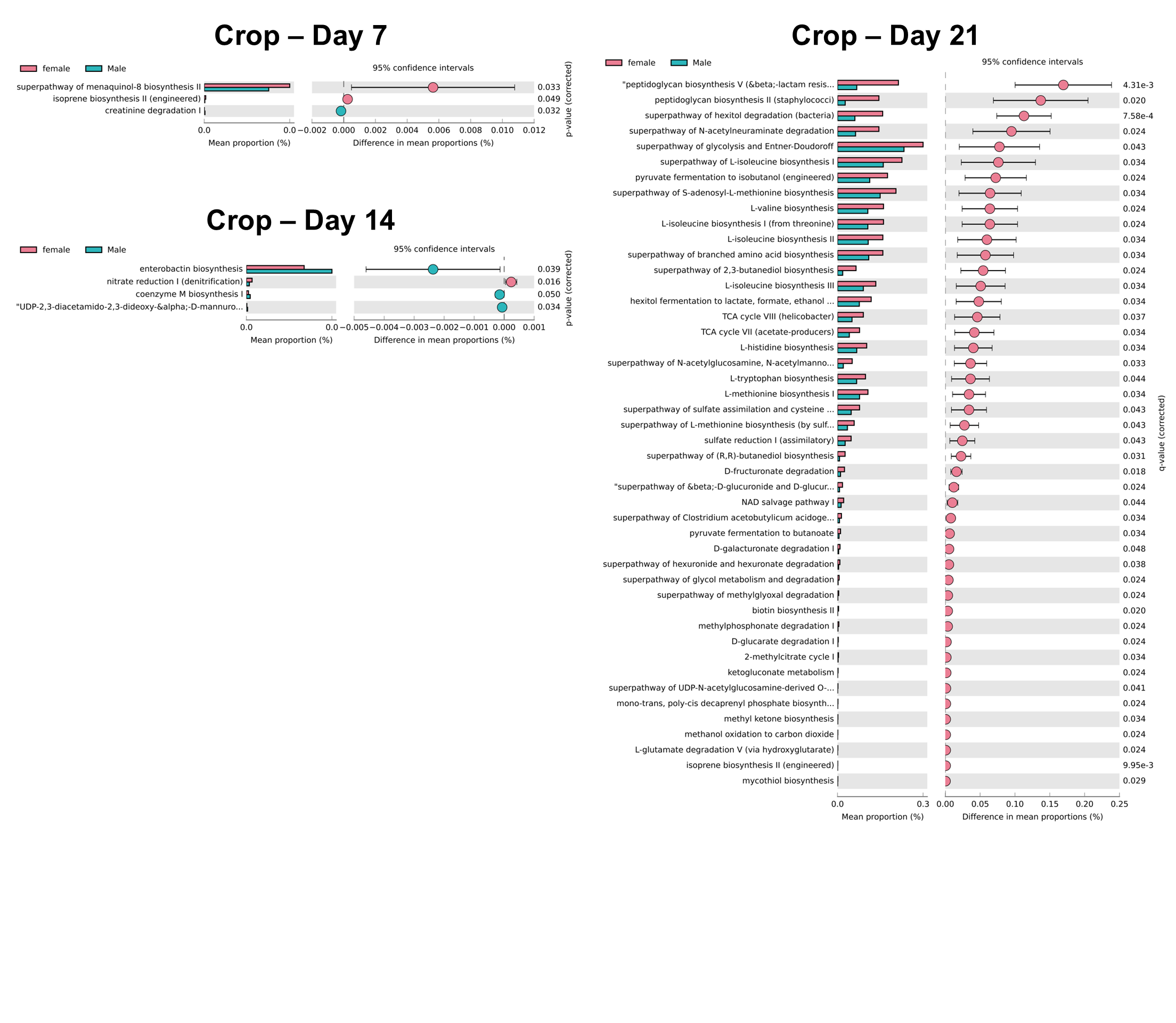


**Fig. S1** Predicted functional pathways differing between male and female broilers in the crop at d 7, 14, and 21, based on PICRUSt2. Differential pathway abundances were identified using Wilcoxon rank-sum tests with Benjamini–Hochberg FDR correction (FDR < 0.05). Bars show the mean relative abundance of significantly enriched pathways, with red indicating enrichment in females and blue indicating enrichment in males; effect sizes are shown on the right. Only pathways meeting the significance threshold are displayed for each gut segment and age.


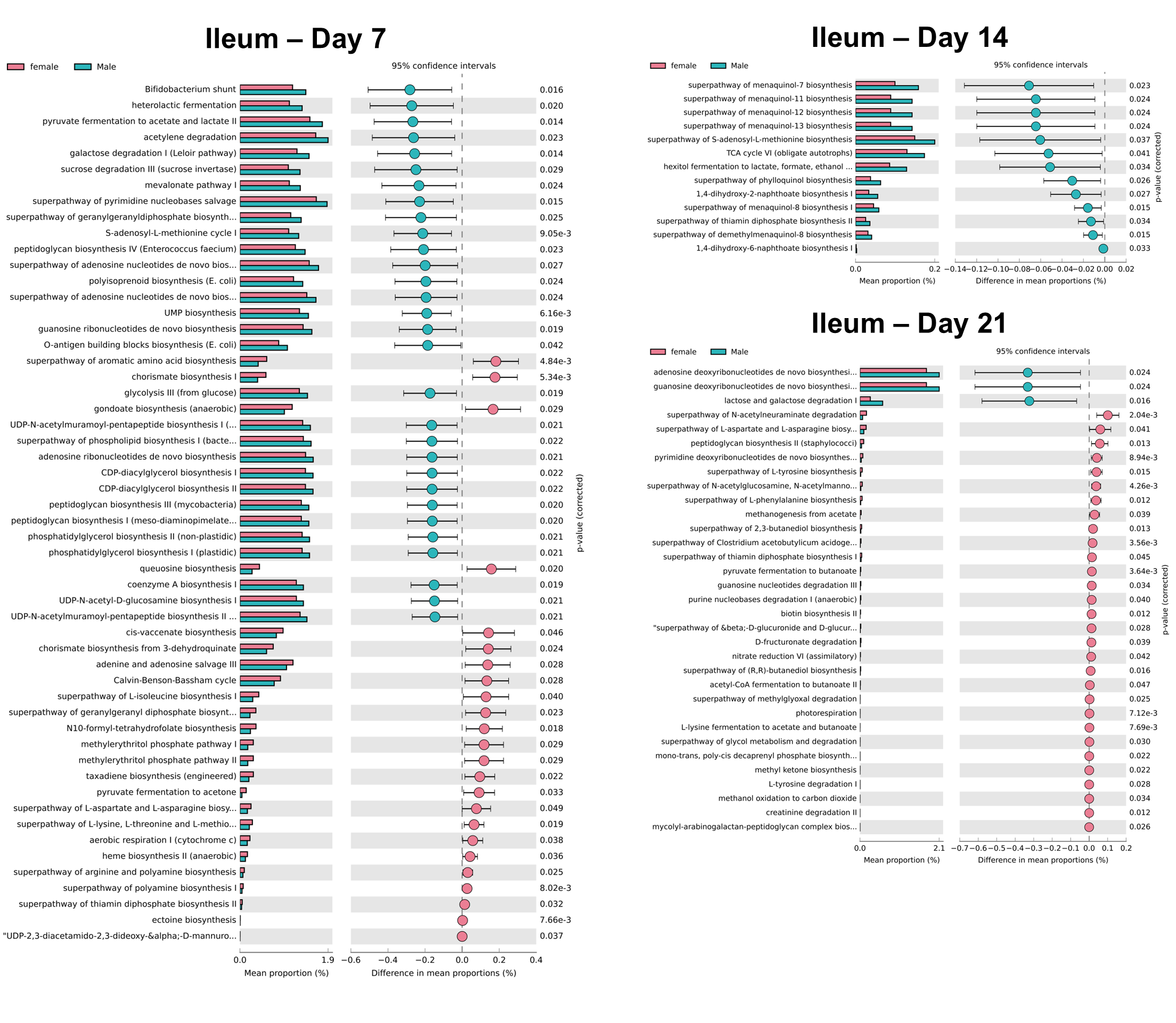


**Fig. S2** Predicted functional pathways differing between male and female broilers in the ileum at d 7, 14, and 21, based on PICRUSt2. Differential pathway abundances were identified using Wilcoxon rank-sum tests with Benjamini–Hochberg FDR correction (FDR < 0.05). Bars show the mean relative abundance of significantly enriched pathways, with red indicating enrichment in females and blue indicating enrichment in males; effect sizes are shown on the right. Only pathways meeting the significance threshold are displayed for each gut segment and age.


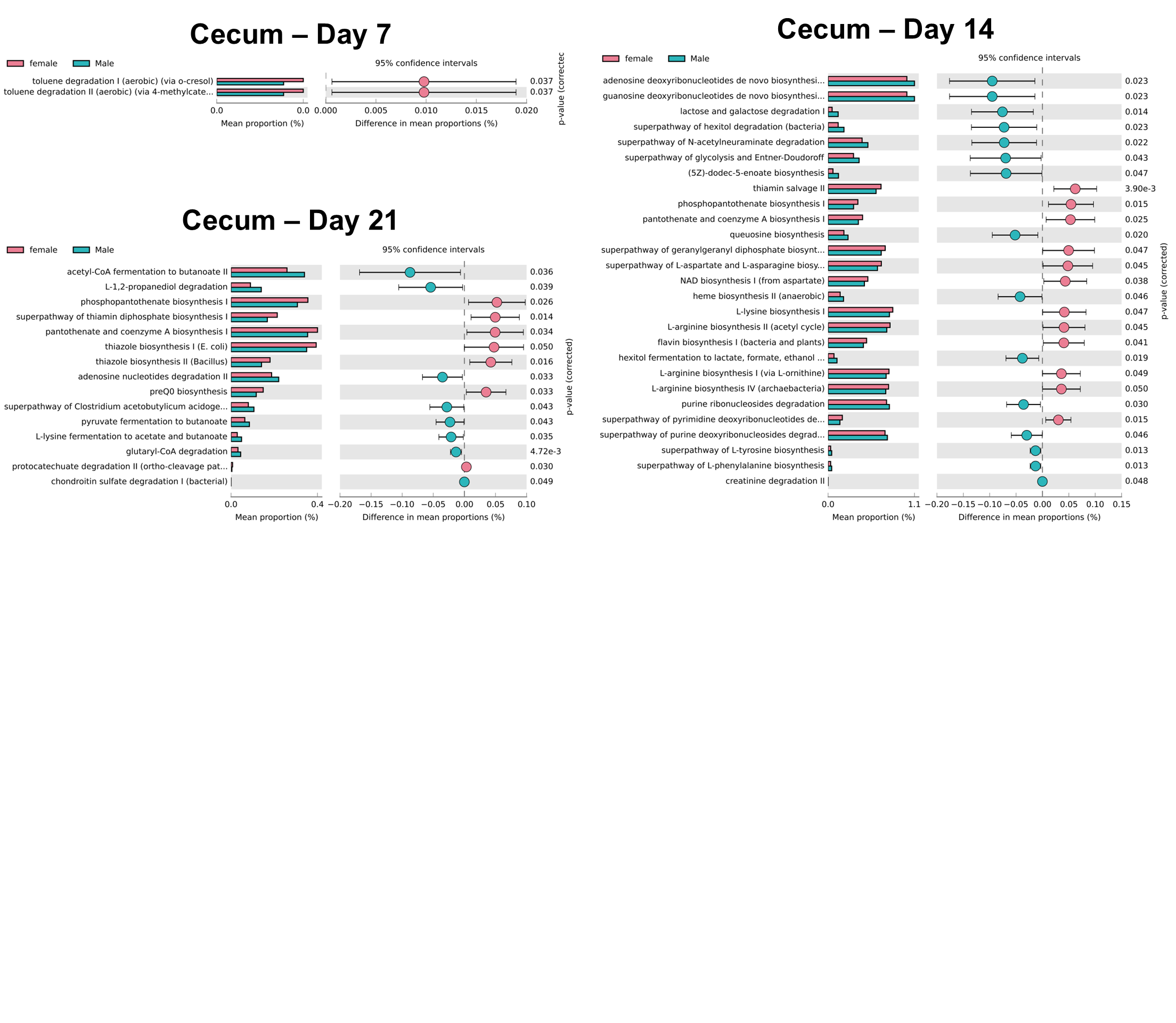


**Fig. S3** Predicted functional pathways differing between male and female broilers in the cecum at d 7, 14, and 21, based on PICRUSt2. Differential pathway abundances were identified using Wilcoxon rank-sum tests with Benjamini–Hochberg FDR correction (FDR < 0.05). Bars show the mean relative abundance of significantly enriched pathways, with red indicating enrichment in females and blue indicating enrichment in males; effect sizes are shown on the right. Only pathways meeting the significance threshold are displayed for each gut segment and age.

**References**

1. Zanu HK, Kheravii SK, Morgan NK, Bedford MR, Swick RA. Interactive effect of dietary calcium and phytase on broilers challenged with subclinical necrotic enteritis: part 2. Gut permeability, phytate ester concentrations, jejunal gene expression, and intestinal morphology. Poult Sci. 2020;99:4914–28. doi:10.1016/j.psj.2020.06.030.

2. Barekatain R, Chrystal PV, Howarth GS, McLaughlan CJ, Gilani S, Nattrass GS. Performance, intestinal permeability, and gene expression of selected tight junction proteins in broiler chickens fed reduced protein diets supplemented with arginine, glutamine, and glycine subjected to a leaky gut model. Poult Sci. 2019;98:6761–71. doi:10.3382/ps/pez393.

3. Konieczka P, Barszcz M, Kowalczyk P, Szlis M, Jankowski J. The potential of acetylsalicylic acid and vitamin E in modulating inflammatory cascades in chickens under lipopolysaccharide-induced inflammation. Vet Res. 2019;50:65. doi:10.1186/s13567-019-0685-4.

4. Hollemans MS, van Baal J, de Vries Reilingh G, Kemp B, Lammers A, de Vries S. Intestinal epithelium integrity after delayed onset of nutrition in broiler chickens. Poult Sci. 2020;99:6818–27. doi:10.1016/j.psj.2020.08.079.

5. Chen X, Naehrer K, Applegate TJ. Interactive effects of dietary protein concentration and aflatoxin B1 on performance, nutrient digestibility, and gut health in broiler chicks. Poult Sci. 2016;95:1312–25. doi:10.3382/ps/pew022.

6. Proszkowiec-Weglarz M, Schreier LL, Kahl S, Miska KB, Russell B, Elsasser TH. Effect of delayed feeding post-hatch on expression of tight junction– and gut barrier–related genes in the small intestine of broiler chickens during neonatal development. Poult Sci. 2020;99:4714–29. doi:10.1016/j.psj.2020.06.023.

7. Forder REA, Nattrass GS, Geier MS, Hughes RJ, Hynd PI. Quantitative analyses of genes associated with mucin synthesis of broiler chickens with induced necrotic enteritis. Poult Sci. 2012;91:1335–41. doi:10.3382/ps.2011-02062.

8. Song Z, Liu L, Sheikhahmadi A, Jiao H, Lin H. Effect of heat exposure on gene expression of feed intake regulatory peptides in laying hens. J Biomed Biotechnol. 2012;2012:484869. doi:10.1155/2012/484869.

9. He X, Lu Z, Ma B, Zhang L, Li J, Jiang Y, et al. Effects of chronic heat exposure on growth performance, intestinal epithelial histology, appetite-related hormones and gene expression in broilers. J Sci Food Agric. 2018;98:4471–8. doi:10.1002/jsfa.8971.

10. Herwig E, Schwean-Lardner K, Kessel AV, Savary RK, Classen HL. Assessing the effect of starch digestion characteristics on ileal brake activation in broiler chickens. PLoS One. 2020;15:e0228647. doi:10.1371/journal.pone.0228647.

11. Zhang JM, Liu XY, Gu W, Xu HY, Jiao HC, Zhao JP, et al. Different effects of probiotics and antibiotics on the composition of microbiota, SCFAs concentrations and FFAR2/3 mRNA expression in broiler chickens. J Appl Microbiol. 2021;131:913–24. doi:10.1111/jam.14953.

12. Criado-Mesas L, Abdelli N, Noce A, Farré M, Pérez JF, Solà-Oriol D, et al. Transversal gene expression panel to evaluate intestinal health in broiler chickens in different challenging conditions. Sci Rep. 2021;11:6315. doi:10.1038/s41598-021-85872-5.

13. Su S, Miska KB, Fetterer RH, Jenkins MC, Wong EA. Expression of digestive enzymes and nutrient transporters in Eimeria acervulina-challenged layers and broilers. Poult Sci. 2014;93:1217–26. doi:10.3382/ps.2013-03807.

14. Borowska D, Rothwell L, Bailey RA, Watson K, Kaiser P. Identification of stable reference genes for quantitative PCR in cells derived from chicken lymphoid organs. Vet Immunol Immunopathol. 2016;170:20–4. doi:10.1016/j.vetimm.2016.01.001.

15. De Boever S, Vangestel C, De Backer P, Croubels S, Sys SU. Identification and validation of housekeeping genes as internal control for gene expression in an intravenous LPS inflammation model in chickens. Vet Immunol Immunopathol. 2008;122:312–7. doi:10.1016/j.vetimm.2007.12.002.
